# Pan‐Cancer Single‐Nucleus Total RNA Sequencing Using snHH‐Seq

**DOI:** 10.1002/advs.202304755

**Published:** 2023-11-27

**Authors:** Haide Chen, Xiunan Fang, Jikai Shao, Qi Zhang, Liwei Xu, Jiaye Chen, Yuqing Mei, Mengmeng Jiang, Yuting Wang, Zhouyang Li, Zihang Chen, Yang Chen, Chengxuan Yu, Lifeng Ma, Peijing Zhang, Tianyu Zhang, Yuan Liao, Yuexiao Lv, Xueyi Wang, Lei Yang, Yuting Fu, Daobao Chen, Liming Jiang, Feng Yan, Wei Lu, Gao Chen, Huahao Shen, Jingjing Wang, Changchun Wang, Tingbo Liang, Xiaoping Han, Yongcheng Wang, Guoji Guo

**Affiliations:** ^1^ Bone Marrow Transplantation Center of the First Affiliated Hospital and Center for Stem Cell and Regenerative Medicine Zhejiang University School of Medicine Hangzhou 310058 China; ^2^ Liangzhu Laboratory Zhejiang University Hangzhou 311121 China; ^3^ School of Biomedical Sciences Li Ka Shing Faculty of Medicine The University of Hong Kong Hong Kong 999077 China; ^4^ M20 Genomics Hangzhou 311121 China; ^5^ Department of Hepatobiliary and Pancreatic Surgery the First Affiliated Hospital Zhejiang University School of Medicine Hangzhou 310006 China; ^6^ Department of Thoracic Surgery Zhejiang Cancer Hospital Hangzhou Institute of Medicine (HIM) Chinese Academy of Sciences Hangzhou 310022 China; ^7^ Department of Laboratory Medicine the First Affiliated Hospital Zhejiang University School of Medicine Hangzhou 310058 China; ^8^ Key Laboratory of Respiratory Disease of Zhejiang Province Department of Respiratory and Critical Care Medicine The Second Affiliated Hospital of Zhejiang University School of Medicine Hangzhou 310009 China; ^9^ Department of Neurosurgery The Second Affiliated Hospital of Zhejiang University School of Medicine Hangzhou 310009 China; ^10^ Department of Colorectal Surgery and Oncology Key Laboratory of Cancer Prevention and Intervention Ministry of Education Zhejiang Provincial Clinical Research Center for Cancer The Second Affiliated Hospital of Zhejiang University School of Medicine Hangzhou 310009 China; ^11^ Zhejiang Key Laboratory of Diagnosis and Treatment Technology on Thoracic Oncology Hangzhou 310022 China; ^12^ Department of Breast Surgery Zhejiang Cancer Hospital Institute of Basic Medicine and Cancer (IBMC) Chinese Academy of Sciences Hangzhou 310022 China; ^13^ Department of Radiology Zhejiang Cancer Hospital Institute of Basic Medicine and Cancer (IBMC) Chinese Academy of Sciences Hangzhou 310022 China; ^14^ Zhejiang Provincial Key Laboratory of Pancreatic Disease the First Affiliated Hospital Zhejiang University School of Medicine Hangzhou 310006 China; ^15^ Zhejiang Clinical Research Center of Hepatobiliary and Pancreatic Diseases Hangzhou 310006 China; ^16^ Key Laboratory of Precise Treatment and Clinical Translational Research of Neurological Diseases Hangzhou 310009 China; ^17^ The Second Clinical Medical College of Zhejiang Chinese Medical University Hangzhou Hangzhou 310053 China; ^18^ The Innovation Center for the Study of Pancreatic Diseases of Zhejiang Province Hangzhou 310006 China; ^19^ Cancer Center Zhejiang University Hangzhou 310058 China; ^20^ State Key Laboratory of Respiratory Disease Guangzhou 510120 China; ^21^ Zhejiang Provincial Key Lab for Tissue Engineering and Regenerative Medicine Dr. Li Dak Sum & Yip Yio Chin Center for Stem Cell and Regenerative Medicine Hangzhou 310058 China; ^22^ Institute of Hematology Zhejiang University Hangzhou 310006 China

**Keywords:** full‐length RNA, high‐throughput and high‐sensitivity, pan‐cancer, single‐nucleus RNA sequencing, total RNA

## Abstract

Tumor heterogeneity and its drivers impair tumor progression and cancer therapy. Single‐cell RNA sequencing is used to investigate the heterogeneity of tumor ecosystems. However, most methods of scRNA‐seq amplify the termini of polyadenylated transcripts, making it challenging to perform total RNA analysis and somatic mutation analysis.Therefore, a high‐throughput and high‐sensitivity method called snHH‐seq is developed, which combines random primers and a preindex strategy in the droplet microfluidic platform. This innovative method allows for the detection of total RNA in single nuclei from clinically frozen samples. A robust pipeline to facilitate the analysis of full‐length RNA‐seq data is also established. snHH‐seq is applied to more than 730 000 single nuclei from 32 patients with various tumor types. The pan‐cancer study enables it to comprehensively profile data on the tumor transcriptome, including expression levels, mutations, splicing patterns, clone dynamics, etc. New malignant cell subclusters and exploring their specific function across cancers are identified. Furthermore, the malignant status of epithelial cells is investigated among different cancer types with respect to mutation and splicing patterns. The ability to detect full‐length RNA at the single‐nucleus level provides a powerful tool for studying complex biological systems and has broad implications for understanding tumor pathology.

## Graphical abstract

In this study, snHH‐seq, a high‐throughput, and high‐sensitivity snRNA‐seq method, is applied to pan‐cancer samples from 32 patients, comprising > 700 000 nuclei from various cancer types. The comprehensive analysis of gene expression, somatic mutations, splicing patterns, and clonal behavior shows broad implications for understanding tumor pathology.

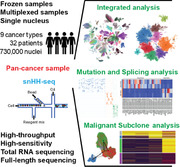

## 1 Introduction

The heterogeneity of cancer cells and their drivers play an important role in tumorigenesis and malignant progression and have significant impacts on cancer treatment.^[^ ^1^ ^]^ Single‐cell RNA sequencing (scRNA‐seq) enables the measurement of transcriptional information at the single‐cell level to accurately resolve tumor heterogeneity. Many studies have used scRNA‐seq to illustrate the diverse tumor microenvironment,^[^ ^2^, ^3^, ^4^, ^5^ ^]^ reveal the mechanism of therapeutic strategies and accordingly propose new molecular markers and therapeutic targets.^[^ ^6^, ^7^ ^]^

However, scRNA‐seq has unique logistical and technical challenges in the processing of clinical tumor samples, especially archival materials (e.g., frozen tissues).^[^ ^8^ ^]^ First, scRNA‐seq of clinical tumor samples requires the implementation of a rapid tissue dissociation program that currently does not exist in the routine pathology laboratories of most hospitals. Second, the majority of scRNA‐seq methods use oligo‐dT primers to capture and amplify polyadenylated transcripts, and this process is highly dependent on the quality of tissue samples. In addition, the oligo‐dT capture results in the absence of non‐polyadenylated transcripts that are important for many biological processes.^[^ ^9^ ^]^ Third, high‐throughput scRNA‐seq methods detect only short fragments of the 3′ or 5′ end of the transcript, which limits the mutation and splicing analysis of clinical samples, especially for tumors.

To overcome these challenges, we developed “high‐throughput and high‐sensitivity single‐nucleus total RNA sequencing” (snHH‐seq), which combines random primers^[^ ^10^, ^11^ ^]^ and a preindex strategy^[^ ^12^ ^]^ in the droplet microfluidic platform. Single‐nucleus RNA‐seq (snRNA‐seq) reduces the requirement for sample collection and processing.^[^ ^13^, ^14^ ^]^ It also paves the way for the analysis of longitudinal samples. Transcript capture using random primers instead of oligo‐dT primers not only rescues archival clinical samples with partially degraded transcripts but also obtains higher sensitivity (one transcript has several sites for capture) and higher coverage (both 3′ and 5′ ends of the transcripts, both non‐polyadenylated and polyadenylated transcripts).^[^ ^15^ ^]^ The preindex strategy is applied in both droplet‐based^[^ ^16^ ^]^ and plate‐based^[^ ^12^ ^]^ methods that offer at least one order‐of‐magnitude gain in throughput and facilitate the multiplexed analysis of clinical samples.

Taking into account the characteristics of the data generated by snHH‐seq, an innovative analysis platform has been established to specifically encompass the entire process from upstream to downstream. This platform tackles key challenges, including quality control of sequencing reads, analysis of full‐length transcripts, and expanding the utilization of total RNA for comprehensive studies. snHH‐seq platform was applied to tumor samples from 32 patients, comprising >700 000 nuclei from various cancer types, including liver, lung, intestine, brain, stomach, esophageal, and breast cancer. This pan‐cancer level sample collection allowed us to conduct a comprehensive analysis, potentially uncovering unique characteristics for each tumor type as well as commonalities shared among different types of cancer. The functions of key cell types and regulatory genes in tumorigenesis and malignant progression were analyzed, including the identification of a pan‐cancer malignant ciliated‐like cell cluster and several transcriptional characteristics of cell proliferation specific to malignant cells. Combining transcriptional information with splicing and mutational information, we detected variants in small nuclear RNAs and their potential splicing effects on cancer markers related to the mechanism of tumorigenesis. Based on the somatic variants and CNV patterns, we also constructed a clonal evolution model for one COAD sample and analyzed malignant progression.

In conclusion, snHH‐seq and its accompanying analysis platform provide a valuable framework for advancing transcriptomic research and analysis, particularly in the context of clinical samples and complex biological processes. This pan‐cancer study enables comprehensive profiling of the transcriptome, covering expression, mutation, splicing, and clone dynamics. A robust pipeline has been established to facilitate the analysis of full‐length RNA‐seq data, and a comprehensive pan‐cancer database has been constructed. This study serves as a foundation for understanding the molecular underpinnings of major cancers associated with high morbidity and mortality in China. Overall, these advancements have significantly advanced our understanding of cancer biology and hold immense potential for improving cancer diagnosis, prognosis, and treatment outcomes.

## 2 Results

### 2.1 snHH‐Seq: A High‐Throughput and High‐Sensitivity Platform for Single‐Nucleus Total RNA Sequencing

**Figure 1:**
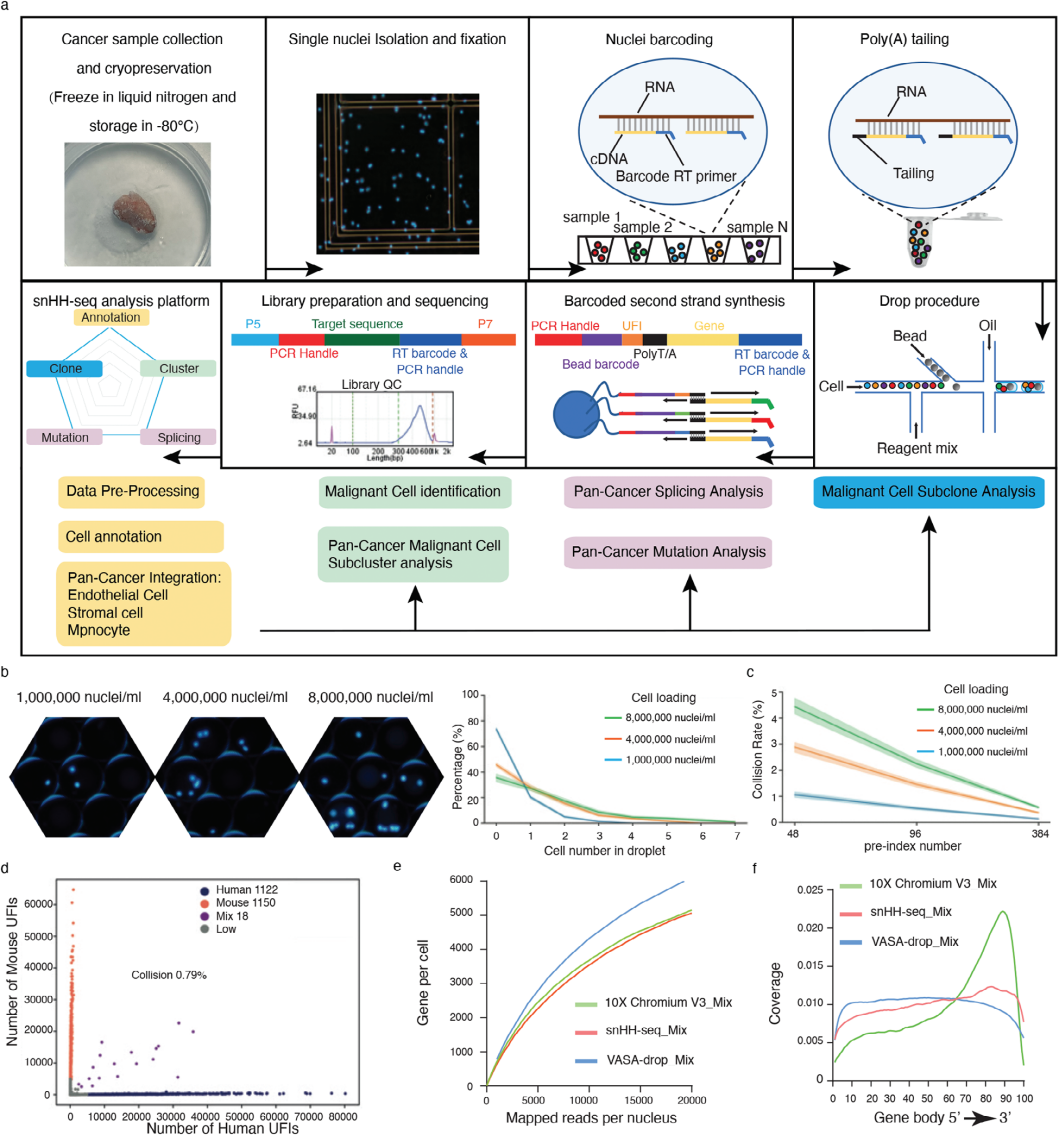
Workflow and evaluation of snHH‐Seq. a) A schematic of the basic workflow for snHH‐seq. b) Nuclei overloading boosts the percentage of droplets filled with nuclei. Nucleus concentration: ≈1000 000 nuclei mL^−1^, 4000 000 nuclei mL^−1^, 8000 000 nuclei mL^−1^. c) Expected collision rate as a function of the nuclei loading concentration for snHH‐seq with different numbers of round1 barcodes. b,c) The line delineates the mean value, and the shading indicates the 95% confidence interval (CI). d) Scatter plot of human‐mouse mix test using snHH‐seq (48 preindex barcodes, load with ≈1000 000 nuclei mL^−1^, 50 µL droplet). Blue dots indicate human‐specific cells; red dots indicate mouse‐specific cells. Only 0.79% (purple dots) are human‐mouse mixed cells. e) Saturation analysis of snHH‐seq, 10X Chromium V3 and VASA‐drop. The number of genes detected per nucleus when downsampling total read counts to the indicated depths. f) Read coverage along the gene body for snHH‐seq, 10X Chromium V3, and VASA‐drop.

To enable the assessment of massive numbers of clinically frozen samples and reduce the batch of dissociation,^[^ ^13^, ^14^ ^]^ we established snHH‐seq, a droplet‐based^[^ ^17^ ^]^ single‐nucleus total RNA sequencing platform with high throughput and high sensitivity (**Figure** **1**a; Figure S1a, Supporting Information). First, tissue was dissociated using liquid nitrogen or scissors, and nuclei were isolated. Then, nuclei were fixed using PFA and barcoded (1st round) in reverse transcription (RT) reactions using well‐specific RT primers. The RT preindex strategy can increase the throughput of single‐cell RNA‐seq and profile massively multiplexed samples in a single experiment.^[^ ^12^, ^16^ ^]^ Random RT primers were used to capture both polyadenylated and non‐polyadenylated transcripts. Moreover, a single transcript was barcoded by several RT primers that increased the sensitivity, and the unique fragment identifier (UFI) was used to quantify the molecules with strand specificity.^[^ ^18^ ^]^ After RT barcoding, nuclei were collected and mixed to add poly(A) tails at the 3′ end of the cDNAs using terminal transferase (TdT). Then, we used the conventional microfluidic platform to produce droplets.^[^ ^17^ ^]^ After preindexing, we overloaded nuclei in the microfluidic chip to increase the throughput (Figure 1b). The collision rate after overloading was controlled by increasing the preindex combination (Figure 1c; Figure S1b, Supporting Information). The preindex strategy promotes parallel analysis of clinical samples. In the droplet, the barcoded oligo‐dT bound with the poly(A) tail of cDNA, and a second barcode was added to cDNA after extending. Finally, we broke the droplets and amplified the cDNA fragments for next‐generation sequencing (NGS).

To assess the fidelity of snHH‐seq, we performed a species‐mixing experiment with cultured human (293T) and mouse (3T3) cells. The size of the cDNA was 200–500 bp (Figure S1c, Supporting Information) and was suitable for NGS without fragmenting. After data processing, we obtained 2290 nuclei (293T: nuclei 1122, median gene 4729; 3T3: nuclei 1150, median gene 3605) and showed that snHH‐seq produced high‐fidelity single‐cell libraries with no more than 0.8% cell doublets (Figure 1d,e; Figure S1d, Supporting Information). Unlike poly(A)‐based 10X Chromium, snHH‐seq exhibited homogeneous coverage across the gene body, similar to VASA‐seq^[^ ^18^ ^]^ (Figure 1f), and made it possible to discover and trace mutations in clinical samples at the single‐cell level. Cryopreserved samples are more convenient for longitudinal and multiplex analysis. We used snHH‐seq with and without the preindex strategy to analyze fresh and frozen mouse brains. The different batches merged well in both UFI/Gene distribution and cell clustering (Figure S1e–h, Supporting Information). The cryopreservation process and preindex process did not significantly alter gene expression and facilitated the analysis of clinically multiplexed samples. In the mouse brain, we obtained 10 546 cells (median UFI 13 094, median gene 3468) and identified 19 cell clusters, including various neurons (Rgs9, Synpo2, etc.), astrocytes (cluster 8/13: Glis3, Slc1a3, etc.), oligodendrocytes (cluster 9: Plp1, Bcas1, Pdgfra, etc.), microglial cells (cluster 17: Inpp5d, Dock2, etc.), and endothelial cells (cluster 16: Rgs5, Flt1, etc.) (Figure S1i,j, Supporting Information). Compared with a brain sample of 10X Chromium, we detected more genes of different types (Figure S1k, Supporting Information), including lincRNA genes, snRNA genes, processed pseudogenes, protein‐coding genes, etc.

We then evaluated snHH‐seq on tumor samples. First, we assessed different buffers for nucleus isolation in tumor samples, including 0.1% IGEPAL CA‐630,^[^ ^19^ ^]^ 0.1% NP40, and Tween with salts and Tris (TST).^[^ ^14^ ^]^ As previously reported, TST showed the highest expression of mitochondrial genes (Figure S2a, Supporting Information). Overall, the three nucleus isolation buffers had comparable performances in terms of UFI/Gene distribution, cell clustering, and cell type diversity (Figure S2a–d, Supporting Information). Second, we assessed the quality of barcoded nuclei after cryopreservation. Both fresh and frozen barcoded nuclei had comparable performances in terms of UFI/Gene distribution, cell clustering, and cell type diversity (Figure S2e–g, Supporting Information). Third, we checked the read distribution of snHH‐seq. Using nuclei for RNA‐seq, snHH‐seq effectively depleted the cytoplasmic rRNAs without an extra rRNA removal step (Figure S2h, Supporting Information). snHH‐seq effectively detected nascent RNA with a high percentage of intron reads (Figure S2i, Supporting Information).^[^ ^10^ ^]^ Fourth, we compared the snHH‐seq data to the Microwell‐seq data. We recovered similar main cell groups in the two methods (Figure S3a,b, Supporting Information) but at different proportions. As previously described, more parenchymal and structural cells were obtained in nuclei dissociation, and more immune cells were captured in cell digestion.^[^ ^14^ ^]^ The stress signature of dissociation was greater in cells (Microwell‐seq) than in nuclei (snHH‐seq) (Figure S3c, Supporting Information). Nuclei profiles had higher levels of long transcripts and transcripts with long poly‐A tails (Figure S3d–f, Supporting Information), consistent with previous reports.^[^ ^13^ ^]^ snHH‐seq detected a higher fraction of protein‐coding transcripts, transcription factors (TFs), lncRNAs, non‐polyadenylated genes,^[^ ^9^ ^]^ and sncRNAs (Figure S3g, Supporting Information). Based on the characteristics of the data generated by snHH‐seq, we also constructed an analysis process to analyze the tumor samples (Figure S3h, Supporting Information).

Overall, snHH‐seq is a powerful approach that combines the advantages of random RT primers and preindex strategy. With high throughput and high sensitivity, snHH‐seq promotes new possibilities for mapping tumor atlases and other genomic studies.

### 2.2 Mapping the Pan‐Cancer Landscape at Single‐Nucleus Resolution

**Figure 2:**
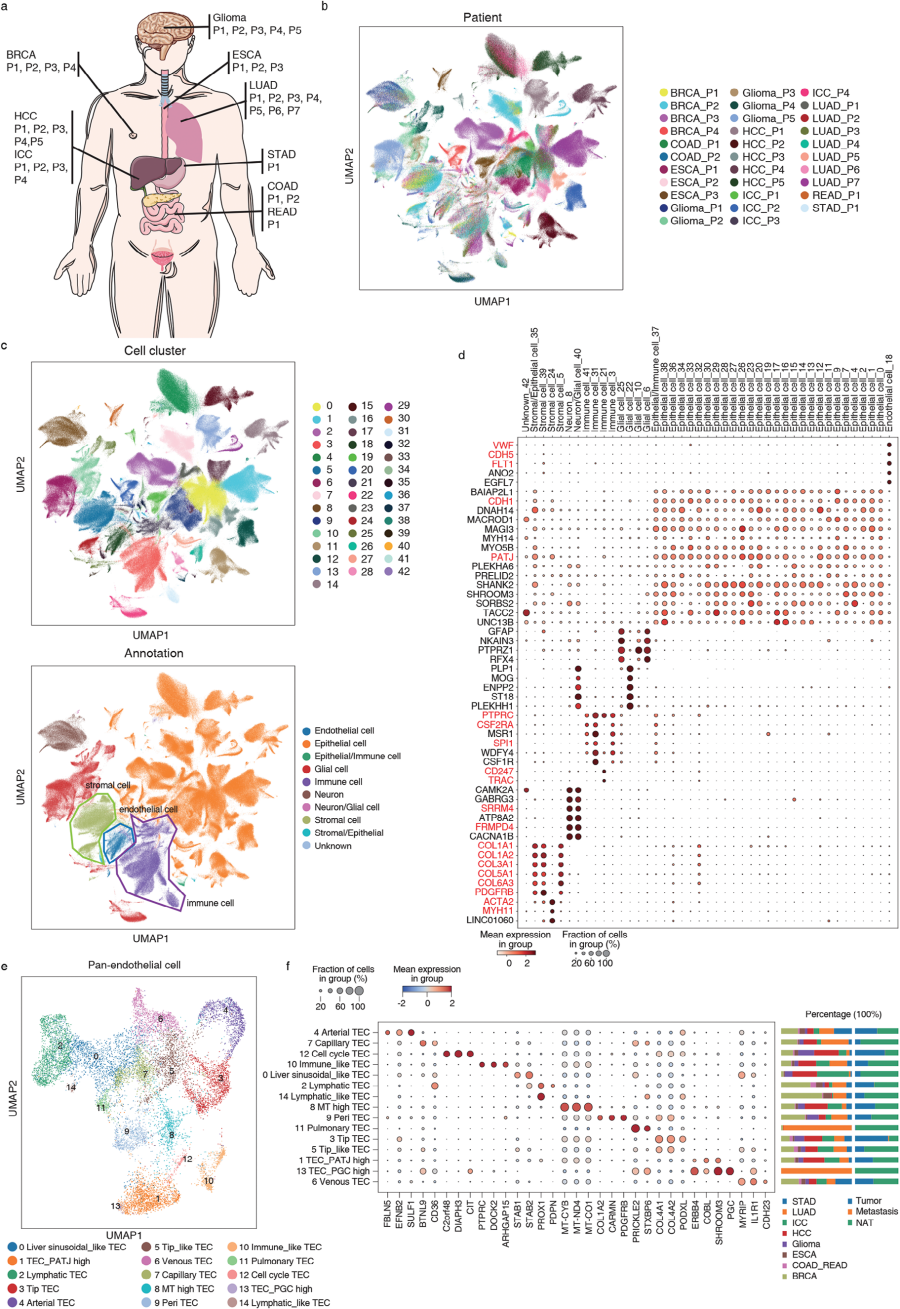
Pan‐cancer analysis using snHH‐seq. a) Schematic indicating tumors collected in this study. b,c) Uniform manifold approximation and projection (UMAP) embedding of cells from the 32 tumors analyzed in this study. Color‐coded for patient b) and cell type c) (up: cell cluster, down: cell type). d) Expression in each cell cluster (columns) of marker genes (rows). e) UMAP of pan‐cancer endothelial cells. f) Marker gene expression per tumor endothelial cell (TEC) cluster. The right bar plot shows the tumor distribution in each TEC cluster. Color‐coded for tumor.

Next, we profiled frozen clinical tumor samples using snHH‐seq. We analyzed 735 722 nuclei from 32 patients spanning tumor types with the highest morbidity and mortality in China, including lung adenocarcinoma (LUAD), hepatocellular carcinoma (HCC), intrahepatic cholangiocarcinoma (ICC), glioma, colon adenocarcinoma (COAD), rectum adenocarcinoma (READ), breast invasive carcinoma (BRCA), esophageal carcinoma (ESCA), and stomach adenocarcinoma (STAD) (**Figure** **2**a; Figure S4a and Table S1, Supporting Information). In single‐cell cancer studies, identifying malignant cells is a crucial step, as they make up the major component of cancer samples. To identify the cell types (malignant cells and nonmalignant cells) in our dataset, we utilized a combination of marker genes and inferred copy number variation (CNV) (https://bis.zju.edu.cn/PCL/ and Table S2, Supporting Information). Taking into account the types of cancer being studied, we used annotated nonepithelial cells, such as endothelial cells, stromal cells, and macrophages, as references for inferCNV to infer the CNV features for each patient.

The complete nuclei dataset was grouped into 43 major clusters (Figure 2b–d; Figure S4b–d, Supporting Information). Each cell cluster was annotated according to the expression of cell type‐specific markers. The 43 cell clusters were divided into six major cell types, namely, epithelial cells (CDH1, PATJ), endothelial cells (CDH5, FLT1, VWF), stromal cells (COL1A1, COL1A2, ACTA2, MYH11), immune cells (PTPRC/CD45, SPI1, CSF2RA/CD116), neurons (SRRM4, FRMPD4), and glial cells (GFAP, PTPRZ1, PLP1). Our analysis revealed that most of the epithelial clusters were patient‐specific, while some epithelial clusters were tumor‐specific with multipatient contributions (C4, C7, C16, C17, C26, C27, C28, C36, and C38). Notably, only C0 and C9 were epithelial cells with multitumor contributions. C0 highly expressed the genes of microvillus organization and embryonic morphogenesis (Figure S4e, Supporting Information). C9 was the epithelium of the digestive tract (esophagus, stomach, and intestine) enriched in gene functions of digestion and cell population proliferation. We also observed that the tumor microenvironment (TME)‐related endothelial cells, stromal cells, and immune cells of different patients were well merged respectively (Figure 2c; Figure S4c, Supporting Information). C5 was the stromal cell with multi‐tumor contributions. Other stromal clusters (C24, C35, C39) were tumor‐specific: C24 cells were from breast cancer and expressed smooth muscle markers; C35 cells were ICC_P2 specific with both epithelial and stromal features, which may be related to epithelial to mesenchymal transition (EMT); C39 cells were brain stromal cells of glioma with multipatient contributions. The immune clusters with multitumor contributions corresponded to macrophages (C3, MSR1) and T cells (C21, CD247, and TRAC), while C31 cells were microglia of glioma with multipatient contributions, and C41 cells were Kupffer cells of ICC with multipatient contributions. This finding suggests distinct characteristics of the TME in different patients and tumors (Figure S4b, Supporting Information) and highlights the importance of considering patient‐specific and tumor‐specific features in cancer research.

To further illustrate the heterogeneity of the TME (tumor microenvironment), we pooled endothelial cells, myeloid cells, and stromal cells from different patients and performed subclustering analyses (Figure 2e; Figure S5, Supporting Information). We obtained 15 subclusters of tumor endothelial cell (TEC), including arterial TEC (C4, FBLN5, and SULF1), venous TEC (C6, ACKR1), lymphatic TEC (C2, PROX1), capillary TEC (C7, BTNL9, and CD36), peri‐TEC (C9, PDGFRB), tip TEC (C3, COL4A1), cell cycle TEC (C12, C2orf48, and CIT), and immune‐like TEC (C10, DOCK2, and PTPRC) (Figure 2f; Figure S5a, Supporting Information). Tip TEC (C3) and cell cycle TEC (C12) were enriched in multiple tumors. In contrast, capillary TEC (C7) mainly resided in normal tissue adjacent to the tumor (NAT).^[^ ^2^ ^]^ In pan‐cancer myeloid cells, we observed shared and specific gene signatures between the tumor and NAT. For example, we obtained M1‐like macrophages (C6/C7) with the expression of IL1B,^[^ ^2^ ^]^ PDE4B,^[^ ^20^ ^]^ MDM2, and PELI1^[^ ^21^ ^]^ in the lung NAT (Figure S5b–e, Supporting Information). In contrast, TAM (tumor‐associated macrophage) C16 was enriched in lung tumors and highly expressed PDE4B but not IL1B, MDM2, or PELI1. Both C1 and C14 had similar expression profiles and expressed PPARG, the resident alveolar macrophage marker.^[^ ^2^, ^22^ ^]^ However, notably, C14 was from lung tumor with low expression of FN1, whereas C1 was from lung NAT and highly expressed FN1 (M2 marker).^[^ ^23^ ^]^ Additionally, several negative regulators were involved in inflammation inhibition in the tumor microenvironment. For example, we detected high expression of RORA,^[^ ^24^ ^]^ a negative regulator of inflammation, in TAM C0 (PTPRF), C10 (PTPRN2), C18 (C2orf48), C20 (WWC1), and C21 (MAGl1), all of which were enriched in tumors; TAM C2 expressed GPNMB,^[^ ^25^ ^]^ a negative regulator of inflammation, and mainly resided in tumors. Furthermore, the cell cycle TAM (C13/18) expressed EZH2, C2orf48, and CIT and mainly resided in tumors. TAM C3 and C17 mainly resided in tumors and coexpressed CD163, MERTK, and LYVE1, which indicated the phagocytosis phenotype.^[^ ^3^ ^]^ In pan‐cancer stromal cells, we obtained 18 subclusters of stromal cells (Figure S5f–i, Supporting Information). Among them, C3 and C6 were myofibroblasts with the expression of DMD. C3 was from the breast tumor with the expression of ENO1 (stress response associated gene), whereas C6 was from both tumor and NAT samples of a variety of tumors. The two matrix CAF (cancer associated fibroblast) subclusters, C4, and C7, exhibited high expression of COL1A1. C7 was enriched in tumors with high expression of COL3A1,^[^ ^26^ ^]^ which may promote tumor metastasis. Another matrix CAF subcluster, C11, was linked to angiogenesis and highly expressed NOTCH3 (an important receptor in vascularization and angiogenesis), COL18A1 (involved in angiogenesis regulation), COL4A1, and COL4A2,^[^ ^27^ ^]^ which might promote the proliferation and metastasis of tumor. Furthermore, we observed significant heterogeneity in breast tumor and NAT samples. C2 and C5 mainly resided in breast tumors and expressed tumor suppressor genes (EXT1^[^ ^28^ ^]^ and HPSE2^[^ ^29^ ^]^), which may regulate the heparan sulfate in the breast TME; C13 mainly resided in breast NAT and highly expressed LRP1B,^[^ ^30^ ^]^ a putative tumor suppressor that may inhibit cancer migration and invasion. The TNXB^[^ ^31^ ^]^ expressing C8 mainly resided in NAT with multi‐tumor contributions and might impede the invasion and metastasis of tumor cells. Overall, our pan‐cancer analysis of the cell compartment revealed shared and cancer‐restricted features of the tumor environment.

### 2.3 Identification of Pan‐Cancer Malignant Cell Subclusters

To investigate the malignant status of epithelial cells among different cancer samples, we first employed CNV analysis to distinguish malignant cells from non‐malignant cells. We merged the inferCNV results from different patients with the same tumor type and performed hierarchical clustering to identify malignant cells in each tissue. Our findings revealed that malignant cells tended to form patient‐specific clusters with relatively high CNV scores (Figure S6a,b, Supporting Information), consistent with the known mechanism of tumor‐related copy number variation. In contrast, nonmalignant cells were successfully discerned, as they tended to form a distinct cluster characterized by relatively low CNV scores, along with the inclusion of multiple patients (Figure S6a, Supporting Information). These findings suggest that nonmalignant cells exhibited less noticeable and significant CNV patterns compared to malignant cells and were thus grouped. We speculated that this difference in CNV patterns between nonmalignant and malignant cells may be due to the underlying genetic mechanisms involved in tumorigenesis. For each tissue, we observed up‐regulated and down‐regulated genes in malignant cells (Figure S6c–e, Supporting Information), which may serve as potential biomarkers or therapeutic targets for cancer treatment. For example, we observed up‐regulated TCF7L1 and down‐regulated DLG5 in BRCA^[^ ^32^, ^33^ ^]^ and up‐regulated DACH1 and down‐regulated SEMA6A in COAD_READ.^[^ ^34^, ^35^ ^]^ Additionally, up‐regulated lncRNA NEAT1 in BRCA^[^ ^36^ ^]^ and down‐regulated lncRNA RP11‐681B3.4 in ESCA, which have not been previously reported, were identified. These results suggest that each type of cancer gained specific expression alterations during tumor progression.

**Figure 3:**
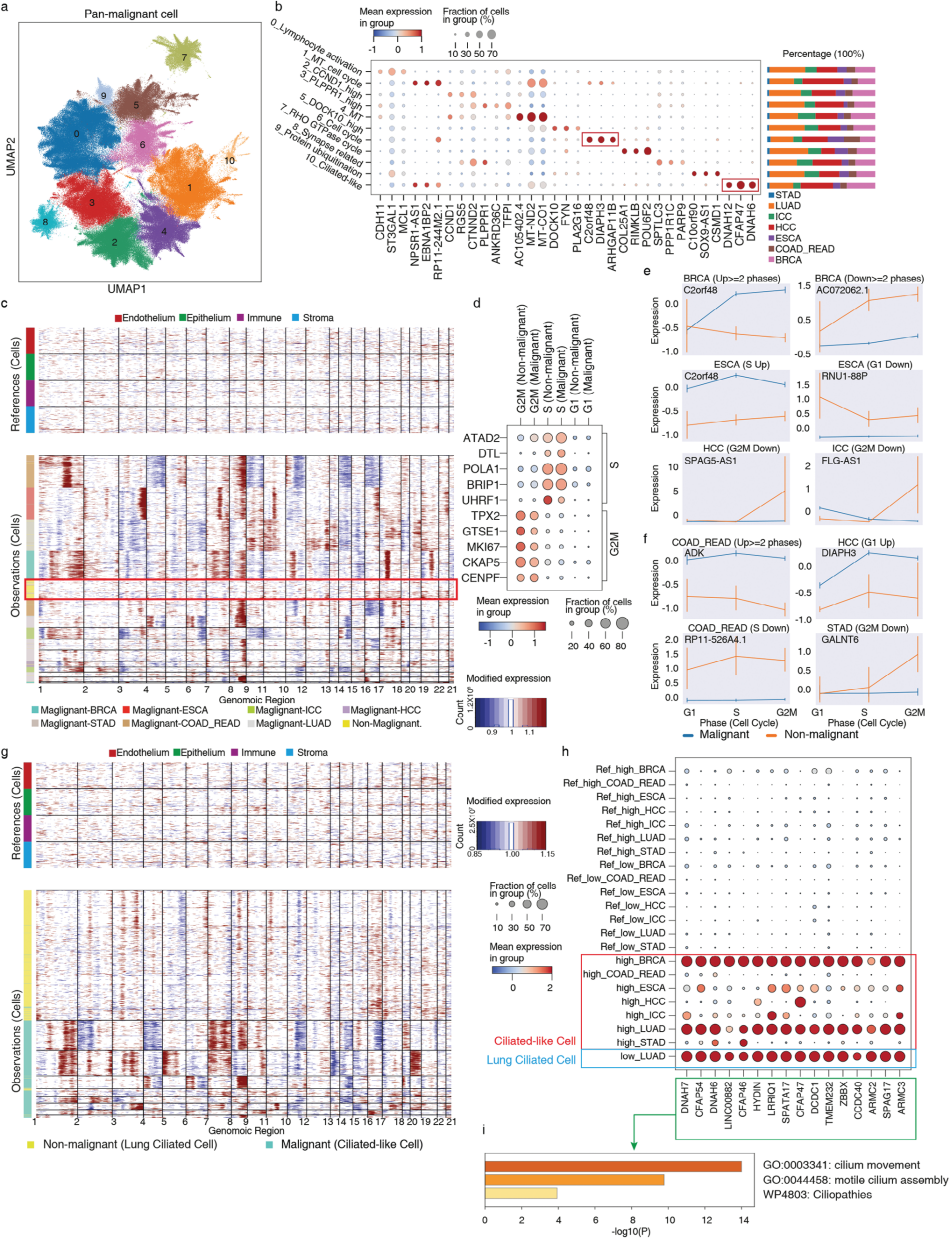
Characteristics of pan‐cancer malignant epithelial cell. a) UMAP of pan‐cancer malignant epithelial cells. b) Marker gene expression and cancer type proportions for each malignant cell subcluster. c) Inferred CNV profiles of proliferating cells. Chromosomal amplification (red) and deletion (blue) are inferred in each chromosomal position (columns) across the single cells (rows). Top: reference cells not expected to contain CNV in tumors. Bottom: proliferating malignant and non‐malignant epithelial cells. Color bar: cell type signature for each cell. d) The expression of cell cycle marker genes in proliferating malignant and non‐malignant epithelial cells. e,f) Expression of selected lncRNA e) and protein‐coding f) genes in proliferating malignant and non‐malignant epithelial cells. g) Inferred CNV profiles of cilia‐related cells. Chromosomal amplification (red) and deletion (blue) are inferred in each chromosomal position (columns) across the single cells (rows). Top: reference cells not expected to contain CNV in tumors. Bottom: malignant ciliated‐like cell and nonmalignant ciliated cell. Color bar: cell type signature for each cell. h,i) The expression of cilia‐related genes in the malignant cell and lung‐ciliated cell with its corresponding GO.

Previous studies have explored gene regulatory modules of malignant cells at a pan‐cancer level by applying NMF on each sample and measuring the similarities between each sample's NMF modules,^[^ ^37^, ^38^ ^]^ and several studies have focused on subtyping malignant cells within specific cancer types by integrating and clustering of malignant cell from several patients.^[^ ^39^ ^]^ However, to date, no studies have examined the commonalities across different cancer types by subtyping malignant cells at a pan‐cancer level. In this study, we addressed this gap by integrating over 300 000 malignant cells derived from seven epithelial‐origin cancers. To eliminate the influence of tissues and cancer types, tissue‐specific genes were removed from the expression matrix, and cancer types were designated as batch labels for Harmony.^[^ ^40^ ^]^ Eleven subclusters of pan‐cancer malignant cells were identified with distinct gene expression profiles (**Figure** **3**a,b). Each cluster was characterized by a specific set of marker genes, which were indicative of the biological processes and pathways active within these cells. C0 exhibited characteristics associated with lymphocyte activation, while C1 displayed gene expression related to both mitochondria and the cell cycle. C2, C3, and C5 were characterized by the upregulation of CCND1 (oncogene), PLPPR1, and DOCK10, respectively. C7 may be associated with the RHO GTPase cycle, and C8 showed upregulation of synaptic genes. C9 may be related to protein ubiquitination. Cells within C4 exhibited a high proportion of mitochondrial transcripts, which may reflect the stress or high energy demand of malignant cells.^[^ ^41^ ^]^ C6 represented a group of proliferating malignant cells commonly observed in tumors. Interestingly, C10 highly expressed cilia‐related genes, which have not been previously discovered. Previous analyses of tumor transcription modules have also revealed that, distinct from other transcription modules, cells expressing proliferating and ciliated‐like transcription modules form separate subclusters.^[^ ^38^ ^]^ Therefore, additional analyses were conducted on C6 and C10.

To capture the intricate transcriptional patterns associated with malignant cell proliferation, we proceeded to conduct further analysis on C6. Compared to proliferating cells from non‐malignant epithelia across different tissues, proliferating malignant cells (C6) exhibited evident CNV features, which was consistent with our previous identification of malignant cells (Figure 3c). Furthermore, we inferred the cell cycle stages of proliferating malignant cells and non‐malignant proliferating cells, assigning them to the G1, S, and G2M phases (Figure 3d). Subsequently, we conducted differential expression analysis and identified a set of coding and noncoding genes that exhibited upregulation or downregulation during different cell cycle stages of malignant cells in each tissue (Figure 3e,f; Figure S7a, Supporting Information). These differentially expressed genes may indicate disruptions in cell cycle control in cancer and provide new markers for cell cycle blockade specific to the cancer cells.

Furthermore, to investigate the cilia‐related signatures across different cancer types, we conducted additional analysis on C10. Despite differences in CNV patterns between C10 malignant cells and lung‐ciliated cells (Figure 3g), C10 malignant cells exhibited high expression of motile cilia‐related genes, including DNAH7 and CFAP54 in BRCA, LUAD, COAD_READ and ESCA; CFAP47 and HYDIN in HCC; DNAH6 and DNAH7 in ICC; CFAP54 and DNAH6 in STAD, similar to those found in lung ciliated cells (Figure 3h,i). Additionally, Figure S7b (Supporting Information) shows the expression proportions and exon proportions of selected cilia‐related genes in non‐malignant lung ciliated cells and ciliated‐like malignant cells, demonstrating consistency and excluding interference from exon proportions. Notably, our results were further confirmed by protein‐level expression of these cilia‐related genes in various types of cancer samples from public databases (The Human Protein Atlas—a tool for pathology) (Figure S7c, Supporting Information). We also observed expression differences between these ciliated‐like malignant cells and nonmalignant lung ciliated cells. For instance, DNAH14, a gene belonging to the DNAH gene family, was up‐regulated in ciliated‐like malignant cells, whereas nonmalignant lung ciliated cells exhibited higher expression of another DNAH family gene, DNAH10 (Figure S7d, Supporting Information). The absence of DNAH10 expression may affect motile cilia assembly, as DNAH10 knockout mice display abnormal sperm flagella structures resembling asthenozoospermia‐like symptoms.^[^ ^42^ ^]^ Additionally, the transcription factor RFX3, associated with lung ciliated cells, was expressed at low levels in ciliated‐like malignant cells (Figure S7c,e, Supporting Information).^[^ ^43^ ^]^ Mutation profiles of ciliated‐like malignant cells and nonmalignant epithelial cells suggest that these expression differences may not be attributed to mutations (Figure S7f, Supporting Information). These results suggest that functional motile cilia may be absent in these malignant ciliated‐like cells. And further research is required to ascertain the presence of cilia structures in these ciliated‐like malignant cells and to explore their specific functions and origins.

### 2.4 Somatic Mutations and Their Effects on Splice Events

**Figure 4:**
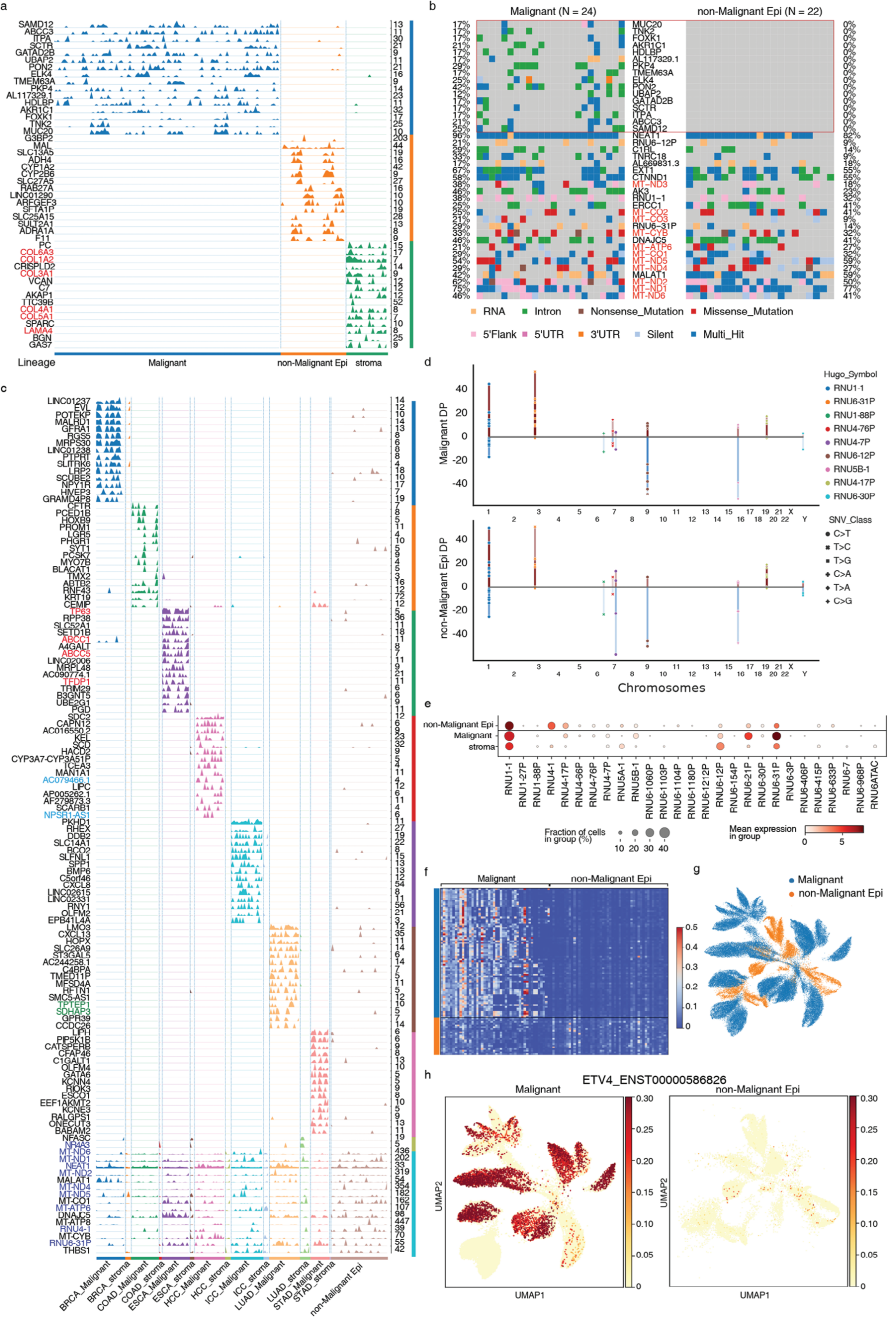
Mutation and splicing characteristics of malignant epithelial cells. a) Commonly mutated genes within three lineages. Blue: malignant cells, Orange: non‐malignant Epithelium, Green: stroma. The numbers on the right indicate the maximum depth of genes. b) Oncoplot of SNV Variants of malignant cells and non‐malignant epithelial cells. 16 common mutations selected by the intersection of mutation count and depth profiles and top mutated genes of malignant cells are shown, colored by mutation classification. c) Commonly mutated genes within cancer types compared the stromal cells and non‐malignant epithelium cells. Different colors correspond to different cancer types. d) Mutation variants of snRNAs and their location in chromosomes, height reflects depth, one point indicates one sample, SNV class is shown in different markers. TOP: Variants in malignant cells; Bottom: Variants in nonmalignant epithelium). e) Variant counts of snRNAs in different lineages. f) Heatmap of cancer marker transcripts ratio between malignant cells and nonmalignant epithelial cells. g) UMAP of inferred CSC transcripts expression of malignant cells and nonmalignant epithelial cells. h) Pseudo transcript expression of ENST00000586826 between malignant cells and nonmalignant epithelial cells.

Understanding the genetic alterations and molecular processes underlying cancer development is crucial for identifying potential therapeutic targets. We performed a comprehensive analysis of transcription mutations and their potential relationship with splicing effects resulting from malignancy among different tumor patients. Nonsynonymous mutations and synonymous mutations were both included as it was previously reported that synonymous mutations might play a role in driving human cancer counts.^[^ ^44^ ^]^ The number of mutation loci per gene (count per gene) per sample provides information about the diversity and distribution of mutations within a gene. The depth of mutations per gene (depth per gene) per sample represents alternative allele count for a specific gene. We calculated these metrics by per patient level or per cluster level to get confidence in the detected mutations and conducted a Wilcoxon test on three groups: malignant group, stromal group, and nonmalignant epithelial group. Mutated genes that exhibited statistical significance with a log‐fold change greater than 20 were selected, and common significant mutated genes of cancer cells by four approaches were detected (**Figure** **4**a,b; Figure S8a,b, Supporting Information). The correlation of mutation among different cancer types was observed.

Mutations in mitochondrial genes were detected in both malignant and nonmalignant epithelial cells. In cancer cells, these mutations can induce functional alterations in mitochondria, including impaired oxidative phosphorylation and disrupted energy metabolism. Additionally, we observed mutations in genes related to the Wnt pathway, including STK3, UBR5, and BICC1, suggesting their potential role in cancer progression. Mutations in ITGB1, TFRC, XPO1, PICALM, PKP4, and MSI2, which are associated with Rho GTPases, were identified. These mutations indicate alterations in cytoskeletal dynamics and cell morphology, affecting crucial cellular processes such as adhesion, migration, and shape changes. Notably, specific mutations were observed in genes involved in lipid localization and transport, including AKR1C1, HDLBP, and ABCC3, suggesting disruptions in lipid metabolism that facilitate the rapid proliferation of cancer cells.

In the stromal group, mutations were found in genes related to the extracellular matrix (ECM). ECM proteoglycans are vital for maintaining tissue and organ integrity, and mutations in ECM‐related genes, such as COL1A2, COL3A1, COL4A1, COL5A1, COL6A3, and LAMA4, can lead to altered ECM composition and organization. Researchers also found prognostic value of extracellular matrix gene mutations in cancers.^[^ ^45^ ^]^ These changes might facilitate the generation of a permissive microenvironment for tumor cells by affecting critical processes such as cell adhesion, migration, and ECM interactions through the PID INTEGRIN1 pathway.^[^ ^46^ ^]^

By randomly sampling 1000 cells from different patients, a mutation count matrix was extracted and PCA components were calculated and used to do correlations (Figure S8c, Supporting Information). To examine the cancer‐type‐specific mutations, we analyzed the variation signature of different cancer types compared with normal groups (Figure 4c). Mutations still appeared to be relatively tumor‐type‐specific. We identified some mutation markers specific to tumor types, including some typical tumor‐related genes (e.g., TP63, ABCC1, ABCC5,^[^ ^47^ ^]^ and TFDP1 in ESCA) as well as numerous noncoding genes (e.g., NPSR1‐AS1^[^ ^48^ ^]^ and AC079466.1^[^ ^49^ ^]^ in HCC) and pseudogenes (e.g., SDHAP3 and TPTEP1 in LUAD). Additionally, there were accumulations of common mutations in mitochondrial genes (MT‐NDs, MT‐ATP6^[^ ^50^ ^]^), small nuclear RNA (RNU4‐1, RNU6‐31P), and tumor‐related genes (NEAT1,^[^ ^51^ ^]^ NR4A3^[^ ^52^ ^]^). This suggests that a significant number of common mutations may have already accumulated in relatively non‐malignant epithelial cells during tumor development, leading to subsequent transcriptional changes.

By analyzing specific variation types of these mutations, the proportion of intronic mutations was particularly high as a result of single‐nucleus full‐length sequencing, over 90% of total variations (Figure S8d,e, Supporting Information). The well‐known cancer‐related pathways showed very strong signals (Figure S8f,g, Supporting Information). We found extensive mutations in some splicing‐related genes, such as RNU1‐1, RNU‐1‐88P, RN6‐12P, and RN6‐30P, with the same nucleotide changes at the same loci in both malignant and epithelial cells (Figure 4d). On the other hand, some nuclear small RNAs, such as RNU6‐21P and RNU6‐31P, had more mutation counts in malignant cells (Figure 4e). RNU genes encode small nuclear RNAs (snRNAs) that are essential components of the spliceosome, the complex responsible for accurate splicing of pre‐mRNA. We hypothesized that these RNU gene mutations could reflect the cell transcription and splicing process and result in novel splice junctions and alter the splicing pattern of multiple genes, such as known cancer markers.

Total RNA sequencing allows us to obtain more detailed information about the expression proportions of different transcripts for genes at the custom cluster level, while conventional 3′ or 5′ terminal sequencing typically provides gene‐level expression information without distinguishing between individual transcript isoforms. All known CSC(cancer stem cell) genes were selected, and the expression of their transcripts based on the distribution of reads was inferred. Using DICEseq for transcript inference allows us to obtain more detailed information about the expression proportions of different transcripts for genes at the custom cluster level. After clustering based on inferred transcript expression of known CSC genes, it was observed that there were significant differences in transcript expression between malignant and nonmalignant cells (Figure 4f,g), and for each cancer type, CSC transcript expression showed distinction as well (Figure S9a,b, Supporting Information). Additionally, upregulated CSC transcripts were identified, such as the transcript of ETV4 (Figure 4h),^[^ ^53^ ^]^ SERPINE2, MALAT1,^[^ ^54^ ^]^ and pre‐mRNA of ANLN (Figure S9c, Supporting Information).^[^ ^55^ ^]^ Known snRNAs are closely related to the splicing process, and specific isoforms have been reported in many cancers.^[^ ^56^, ^57^ ^]^ Taken together, we believe that the mutations in snRNAs may directly affect the splicing in malignant cells and promote the expression of certain CSC gene marker isoforms.

Overall, we found lots of genetic mutations in cancer cells, which impact various vital signaling pathways, cellular processes, metabolic functions, and splicing patterns. The identification of mutations in snRNAs and splicing isoforms emphasizes the critical role of splicing dysregulation in tumor development and progression. Cancer‐related alterations in the noncoding component of the spliceosome need to be further examined, and detailed functional analysis is required to confirm the role of these isoforms, which will contribute to a more comprehensive understanding of cancer biology and aid in the development of targeted therapeutic strategies.

### 2.5 Copy Number Variation and Somatic Mutational Events Underlie Tumor Metastasis

Tumor metastasis is a complex process that plays a pivotal role in cancer progression and is responsible for the majority of cancer‐related deaths. In this study, we conducted a clonal study on a metastatic COAD sample to understand the patterns and dynamics of metastatic. The sample was obtained from the right‐sided colon cancer of a patient. The pathological type is moderately differentiated adenocarcinoma, with ≈50% of the tumor being mucinous adenocarcinoma. The tumor has infiltrated beyond the serosa and shows evidence of lymphovascular invasion and perineural invasion. Additionally, metastatic adenocarcinoma is observed in the submucosal layer extending to the serosal layer. Among the pericolic lymph nodes examined, one out of twelve shows positive involvement. Invasive or metastatic adenocarcinoma was found in the omental tissue.

In order to examine the clonal characteristics of cancer metastatic, we utilized population‐based CNV methods called Numbat to perform a clonal study on a metastatic COAD sample. Signals from gene expression and SNPs of this COAD sample, and population‐derived haplotype information are integrated to accurately infer allele‐specific CNV to reconstruct their clonal relationship. Expression‐based methods for inferring CNV rely on the assumption that amplifications or deletions in the genome will lead to corresponding changes in gene expression levels. Population‐based phasing leverages the haplotype information from a population reference panel to computationally infer the phase of variants within an individual's genome.

Population‐derived haplotype information is based on the 1000 Genome hg38 SNP VCF file, 1000 Genome hg38 phasing panel file, and Eagle2 hg38 genetic map. The BAM file is used to get SNPs. SNPs were piled up and phased using Cellsnp‐lite and Eagle2, the default settings of Numbat were used for the majority of the analysis, except for two specific parameters: minMAF and minCOUNT. minMAF stands for the minimum minor allele frequency, and SNPs with a minor allele frequency below 0.1 are considered less informative or potentially unreliable. minCOUNT represents the minimum read count, and by setting it to 20, SNPs with a read count below this threshold considered as a result of sequencing errors or low coverage were excluded. Subclones were then inferred based on CNV scores and four subclones were detected.

**Figure 5:**
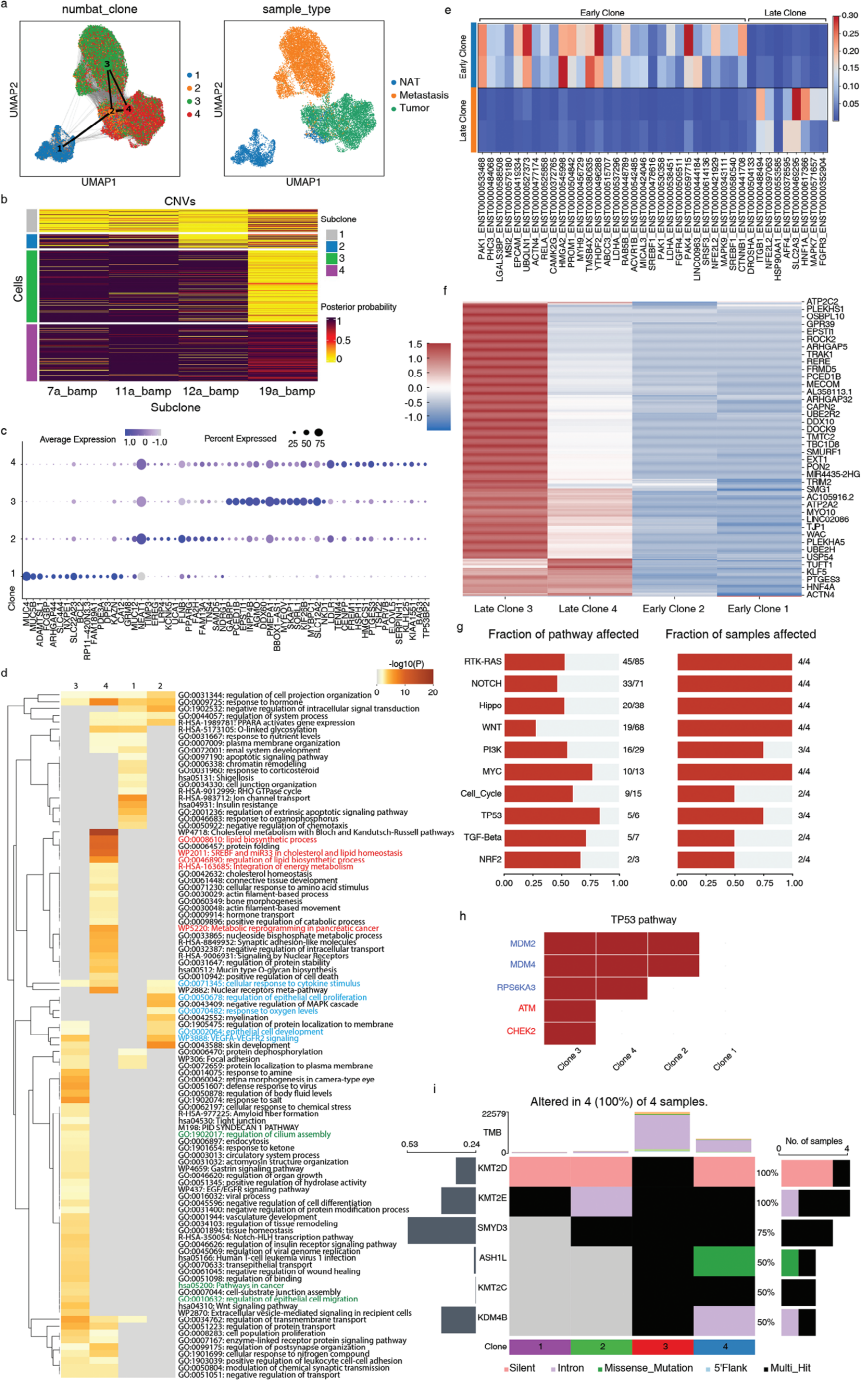
Clone analysis of COAD sample. a) UMAP of COAD sample, cluster connectivity indicated by PAGA. Left: Color of Numbat Clones. Right: Color of Sample types. b) Numbat inferred CNV profiles for 4 clones colored by posterior possibility, balanced amplifications (bamp) on chromosomes 11, 12, and 19 were detected as key events contributing to the subclone lineages. c) Differential expression of 4 Numbat clones. d) Gene ontology (GO) enrichment of four inferred clones using Metascape, top100 GOs are shown. e) Heatmap of cancer marker inferred transcripts ratio between early clones (Clones 1 and 2) and late clones (Clones 3 and 4). f) Profiles of mutation counts among 4 clones, values were z‐score normalized to 0. g) Well‐known pathway affected in COAD sample in terms of the fraction of affected genes and fraction of affected samples. h) TP53 pathway and affected mutated genes, red color indicates tumor suppressor gene. i) Epigenetic mutation occurring in clones.

By utilizing PAGA on the expression matrix, we were able to examine the developmental connectivity and branching patterns of the identified clones, considering the cellular neighborhoods and their connections. Based on the prior knowledge of where the cells were extracted (NAT, tumor, or metastasis), we can compare the inferred clone with the malignancy of cells and get a better understanding of cancer metastasis. Our analysis revealed the correlation between the different samples (NAT, primary tumor, and metastatic tumor) and the identified clones (**Figure** **5**a). Clone 1 may represent a precursor or non‐malignant population, while clones 2, 3, and 4 may be associated with the development and progression of the primary tumor. Clone 2 served as a connection point as an intermediate state cluster. Clones 3 and 4 appear to have a specific role in the metastatic process, as evidenced by their dominance in the metastatic sample.

Upon further examination of the CNV heatmap, we were able to identify balanced amplification (BAMP) on chromosomes 11, 12, and 19 as the key events contributing to subclone lineages (Figure 5b). Amplification of chromosome 12 was observed in various cancer types and has been implicated in tumor progression and aggressiveness.^[^ ^58^, ^59^ ^]^ This amplification event was also found to be a distinguishing feature between late clones (3 and 4) and early clones (1 and 2). To gain a better understanding of these subclones, we performed differential expression analysis on subclones and conducted Gene Ontology (GO) analysis on marker genes (Figure 5c,d). As an intermediate state, marker genes of clone 2 were associated with the regulation of epithelial cell proliferation and response to oxygen levels. The cells in clone 2 may be undergoing alterations in their proliferative capacity and adapting to changes in oxygen availability, which are common features of malignant transformation. The presence of cellular response to cytokine stimulus in clone 2, 3, and 4 suggests an active interplay between the tumor microenvironment and cancer cells. Clone 4 enriched genes of lipid metabolism, which confers the aggressive properties of malignant cancers.^[^ ^60^ ^]^ Particularly, we found regulation of epithelium cell migration and cilium assembly in clone 3, which appeared more in the metastatic sample. The association of cilium assembly regulation with clone 3, particularly in the metastatic sample, suggests a potential role for primary cilia in promoting the migratory and invasive properties of malignant cells. In addition to changes in expression level, specific alternatively spliced transcripts were found for late clones as well, such as ENST00000469295 of SLC2A3 (Figure 5e).^[^ ^61^ ^]^ The presence of specific alternatively spliced transcripts in the late clones, corresponding to previous splicing analysis, suggested that distinct isoform level expression provided better malignant cell signature as they may have distinct functional properties or regulatory mechanisms compared to the early clones.

Our mutation analysis of different subclones revealed notable differences in the mutation profiles. We observed that clones 3 and 4 showed a significant increase in the number of mutations compared to clones 1 and 2, indicating a higher mutational burden in malignant subclones (Figure 5f). The well‐known cancer pathways affected can be observed in multiple subclones (Figure 5g). Specifically, we observed that clone 3 had a higher frequency of tumor suppressor gene mutations within the TP53 pathway than clone 4 (Figure 5h). Mutations in the ATM and CHEK2 have been associated with the development of various cancers, including breast cancer, lung cancer, pancreatic cancer, prostate cancer, and colorectal cancer.^[^ ^62^, ^63^ ^]^ The presence of ATM and CHEK2 mutations in the metastasis clone suggests potential implications for the metastatic process. These mutations may confer selective advantages to cancer cells, allowing them to evade DNA damage checkpoints and acquire characteristics that promote metastasis, such as increased proliferation, survival, and resistance to therapy. In addition, we identified mutations in epigenetic regulation genes,^[^ ^64^ ^]^ such as KMT2D, KMT2E, SMYD3, ASH1L, KMT2C, and KMD4B, in the tumor clones (Figure 5i). These findings suggested potential alterations in chromatin structure and gene regulation processes. Mutations in KMT2D have been identified in various cancer types and are associated with altered gene expression profiles and disrupted cellular differentiation.^[^ ^65^ ^]^ KMT2E is another member of the KMT2 family of histone methyltransferases. Although its precise role in cancer is still being elucidated, emerging evidence suggests that KMT2E may play a role in cell cycle regulation and tumor progression.^[^ ^66^ ^]^ Notably, SMYD3, which accumulated in clone 2, is a histone methyltransferase that contributes to the regulation of gene expression through histone methylation.^[^ ^67^ ^]^ It has been shown to promote tumor growth and metastasis by altering gene expression patterns and affecting signaling pathways involved in cell proliferation and invasion. In addition, late clones were observed to have mutations specifically in ASH1L, KMT2C^[^ ^68^ ^]^ and KMD4B.^[^ ^69^ ^]^ The accumulation of mutations in these epigenetic regulatory genes, particularly in late clones, suggests that these modifications may provide tumor cells with selective benefits, allowing them to bypass growth and survival hurdles.

These results provide insights into the clonal evolution of tumors, highlighting that a comprehensive understanding of tumor metastasis requires a multidimensional analysis that goes beyond gene expression levels. By integrated analysis of CNV, expression, mutations, splicing, and potential epigenetic alterations, researchers can gain a more comprehensive understanding of the molecular landscape of metastatic tumor cells. Future research needs to consider these various aspects and explore the interconnections between them, as they collectively contribute to the metastatic phenotype.

## 3 Discussion

In this study, we present a high‐throughput and high‐sensitivity platform, snHH‐seq, for single‐nucleus total RNA sequencing. The preindex strategy was employed to increase the throughput and facilitate the multiplexed analysis of clinical samples. The use of random primers increases the sensitivity of nucleus‐based analysis and lowers the requirement for sample collection and storage. Additionally, snHH‐seq is compatible with commonly used single‐cell sequencing methods, making it accessible to laboratories worldwide. Supported by the throughput and sensitivity of snHH‐seq, we profiled the transcriptome of cryopreserved pan‐cancer samples and formed a single‐cell transcriptome database to reveal the clinically relevant cell types in tumors.

In essence, RNA‐seq is a primer‐based detection method (both probe and RT primer). The choice of primer determines which transcripts we can detect in a cell. In this study, we used random primers to capture total RNA in nuclei thus allowing the analysis of more genes of different types, particularly lncRNA, snRNA, and pseudogene. We also found that the read distributions form the peaks and exhibit a kind of RNA accessibility, which may be due to PFA crosslinking or uneven distribution of the bases on the genome. So, there is room for optimization in capturing primers and fixing reagents.

The preindex strategy by in situ RT has been applied in several studies. In snRandom‐seq,^[^ ^11^ ^]^ five barcodes were used to reduce the contamination rate of the species‐mixing experiment without overloading, thereby reducing the requirement for the stability of the microfluidic device. The scifi‐RNA‐seq assay^[^ ^16^ ^]^ increases the throughput and facilitates the multiplexed analysis. When compared with scifi‐RNA‐seq, our preindexed high‐throughput method does not require the inefficient ligation step, and thus ensures both throughput and detectability. Moreover, the preindex is not as simple as adding a barcode on RT primer. We observed that excessively long primers (with PCR/ligation handle, barcode, and RNA capture oligo) reduce the efficiency of in situ reverse transcription, which may explain the low gene number in scifi‐RNA‐seq. So, we used bulk in situ RT to screen ≈800 barcodes and obtained 136 barcodes with high efficiency of reverse transcription. Overloading nuclei in microfluidics increases cell throughput, and also poses new challenges to the conventional microfluidic platform. In future research, we will optimize the chip design and density gradient concentration to reduce nuclear adhesion and sedimentation during nuclei overloading, thereby improving nuclei recovery rate.

snHH‐seq provides a novel and comprehensive analytical repertoire for pan‐cancer investigations. Our study analyzed a total of 735 722 nuclei obtained from 32 patients across nine different tumor types. The nuclei were then clustered into 43 distinct cell clusters, which were further categorized into six major cell types: epithelial cell, endothelial cell, stromal cell, immune cell, neuron, and glial cell. To gain a deeper understanding of TME heterogeneity, we performed subclustering analyses on endothelial cells, myeloid cells, and stromal cells from different patients.

In addition to the general analyses above, we conducted a novel investigation into the malignant status of epithelial cells in various cancer samples. No prior studies have delved into the commonalities across different cancer types by subtyping malignant cells at a pan‐cancer level. By integrating data from over 300 000 malignant cells derived from seven epithelial‐origin cancers, we captured the intricate transcriptional patterns associated with malignant cell proliferation. Our findings, particularly the identification of several malignant cell proliferation‐specific ncRNA, contribute to the advancement of this field. Additionally, we found malignant cells with high expression of cilia‐related genes. Ciliated‐like malignant cells have been observed in tissues that contain normal ciliated cells.^[^ ^38^ ^]^ However, the presence of ciliated‐like malignant cells in tissues lacking normal ciliated cells, which has not been reported previously, suggests that ciliated‐like malignant cells may represent a novel subcluster emerging during tumorigenesis or progression. Further investigation is required to not only validate their presence at the protein level but also elucidate their origin and function in cancer. Overall, our study provides valuable insights into the mechanisms underlying malignant cell behavior, which could ultimately lead to improved cancer detection and treatment strategies. Future studies could leverage the availability of public datasets to cluster and investigate malignant cell subclusters at a larger scale, such as the inclusion of thousands of datasets.^[^ ^70^ ^]^

Total RNA sequencing of snHH‐seq presents an opportunity to explore the somatic mutations and copy number variations in cancer development and progression, affecting vital signaling pathways, cellular processes, metabolic functions, and splicing patterns. Notably, mutations in snRNAs and splicing isoforms have been identified, highlighting the significance of splicing dysregulation in tumorigenesis. Moreover, to gain insight into tumor metastasis, we conducted a clonal study on a metastatic COAD sample and identified four subclones. The accumulation of mutations in epigenetic regulation genes, particularly in late clones, suggests that these alterations may confer selective advantages to tumor cells, allowing them to overcome barriers to growth and survival. These mutations may provide cells with increased adaptability, enhanced invasive potential, or resistance to therapy, thereby contributing to the aggressiveness and metastatic behavior of the tumor. Understanding the role of epigenetic dysregulation in tumor clones can provide insights into potential therapeutic targets and strategies aimed at restoring normal epigenetic control and inhibiting tumor progression.

In summary, we demonstrate the value of high‐resolution single‐cell full‐length transcriptomic sequencing for identifying novel tumor biomarkers and provide a detailed analysis pipeline for full‐length single‐cell sequencing. Our comprehensive analysis approach involves examining gene expression, somatic mutations, splicing patterns, and clonal behavior. By leveraging these diverse perspectives, we can identify specific variants that are linked to cancer‐associated genes and cell types. This project represents a significant advancement in our ability to understand the underlying genetic mechanisms across cancers. Ultimately, these findings deepen our understanding of tumor biology and pave the way for more effective diagnostic and therapeutic approaches.

## 4 Experimental Section

### Ethics Statement

The collection of human samples and research conducted in this study was approved by the Research Ethics Committee of the First Affiliated Hospital (approval numbers: IIT20210078B), the Second Affiliated Hospital (approval numbers: IR2022519), and Zhejiang Cancer Hospital (approval numbers: IRB‐2022‐642). Informed consent for collection and research using surgically removed adult tissues was obtained from each patient before the operation. Details on donor information are provided in Table S1 (Supporting Information). All the protocols used in this study were in strict compliance with the legal and ethical regulations of Zhejiang University School of Medicine and Affiliated Hospitals. All the protocols used in this study complied with the “Interim Measures for the Administration of Human Genetic Resources” administered by The Ministry of Science and Technology and The Ministry of Public Health. Mouse experiments in this study were approved by the Animal Ethics Committee of Zhejiang University (ZJU20210079); experiments conformed to the regulatory standards at Zhejiang University Laboratory Animal Center.

### Nuclei Suspension Preparation (Culture Cell)

HEK293T and NIH/3T3 cells were cultured in Dulbecco's Modified Eagle Medium (DMEM, Gibco) supplemented with 10% Fetal Bovine Serum (FBS, Thermo) and 1% penicillin‐streptomycin (Gibco). Cells were cultured in the six‐well culture plates (Corning) in an incubator with humidified air and 5% CO_2_ at 37 °C and passaged every 2–3 days using 0.25% Trypsin‐EDTA (Gibco). Cells were harvested by trypsinization and washed twice using cold Dulbecco's Phosphate‐Buffered Saline (DPBS, Corning). Then 2 × 10^6^ mixed cells (1:1, HEK293T and NIH/3T3) were resuspended in 2 mL ice‐cold Lysis Buffer (LB, 0.1% IGEPAL CA‐630 (Sigma), 1% RNA Inhibitor (Vazyme), 0.1% Tween‐20 (Diamond), 10 mm Tris‐HCL pH 7.5 (Thermo), 10 mm NaCl (Sangon), 3 mm MgCl_2_ (Sigma) in ddH_2_O). The lysis was performed on ice for 5 min. The nuclei were centrifuged at 500 g for 5 min and resuspended in 2 mL ice‐cold Wash Buffer (WB, 1% RNA Inhibitor, 0.1% Tween‐20, 10 mm Tris‐HCL pH 7.5, 10 mm NaCl, 3 mm MgCl_2_ in ddH_2_O). Nuclei were washed twice with WB and resuspended with 5 mL 4% PFA (RNase Free). Nuclei were incubated on ice for 30 min. Then 750 µL 2.5 m Glycine (Diamond) was added to quench the reaction and the mixture was placed on ice for 10 min to stop cross‐linking completely. The fixed nuclei were washed twice using WB and filtered through a 10‐µm strainer to remove clumps. The nuclei were processed to single nuclei RNA‐seq according to the following snHH‐seq protocol.

### Nuclei Suspension Preparation (Tissue Cell)

Frozen human tumor samples were requested from the First Affiliated Hospital of Zhejiang University, the Second Affiliated Hospital of Zhejiang University, and Zhejiang Cancer Hospital, with the sample information summarized in Table S1 (Supporting Information). Wild‐type C57BL/6J male mice (6–8 weeks) were ordered from Shanghai SLAC Laboratory Animal Co., Ltd. All mice were housed at Zhejiang University Laboratory Animal Center in a Specific Pathogen Free facility with individually ventilated cages. The mouse brain was collected and washed in ice‐cold DPBS. Tissues were dissociated by smashing in liquid nitrogen and suspended in LB. The lysis was performed on ice for 5 min and the suspension was then filtered through a 10‐µm strainer to remove clumps. The nuclei were washed and fixed as mentioned above.

### Microfluidic Device Design and Fabrication

The microfluidic devices were designed and custom‐made by M20 Genomics company based on the work by Zilionis et al.^[^ ^17^ ^]^ The channel depth of devices is 50 µm for cell encapsulation, and 30 µm for hydrogel beads. Microfluidic devices were fabricated using polydimethylsiloxane (PDMS) according to the protocol described.^[^ ^71^ ^]^ The equipment of the microfluidic platform includes microfluidic devices, three syringe pumps (Pump 11 Elite 4500 and 4501), a syringe (BD Luer‐lok Tip, 1 mL), and Micro Medical Tubing (Scientific Commodities, I.D. x O.D. x L: 0.015′’ x 0.043′’ x 100 feet.), and an inverted bright‐field microscope with a fast speed camera (FLIR) and a computer.

### Barcoded Bead Synthesis for snHH‐Seq

As previously described,^[^ ^11^ ^]^ the barcoded hydrogel beads were produced by M20 Genomics company. Hydrogel beads were synthesized by the microfluidic emulsification and polymerization of an acrylamide‐primer mix. In this study, the acrydite‐modified oligonucleotides contain a deoxy Uridine base. Therefore, the primers can be released to capture the cDNA by USER enzyme (NEB). After hydrogel bead production, three rounds of split‐and‐pool ligation in the 96‐well plate were performed for barcoding (Table S3, Supporting Information).^[^ ^11^ ^]^ The ligation mixture, including hydrogel bead, DNA ligase (Vazyme, 350 U mL^−1^), 1 x T4 buffer, and nuclease‐free water was prepared and then split into a round‐bottom 96‐well plate. The hydrogel beads were mixed with 96 annealed unique barcode primers (Bead_ligation_1F/R, Bead_ligation_2F/R, Bead_ligation_3F/R) in 96‐well plate, respectively, then incubated at 37°C for 30 min. All the required reagents for hydrogel barcoded beads synthesis and the ready‐to‐use hydrogel barcoded beads can be ordered from M20 Genomics company.

### snHH‐Seq Procedure

The fixed nuclei were counted and suspended in a reverse transcription mixture (RT mix). For a 96‐well plate reaction, 110 x RT mix was prepared: 55 µL 10 mm dNTP, 484 µL RT buffer, 55 µL RNA Inhibitor (Vazyme), 55 µL Reverse Transcriptase, 341 µL WB (with nuclei). The reverse transcription kit was included in the VITAPilote‐EFT1200 kit (Cat # R20122124) ordered from M20 Genomics. Both nuclei‐RT mix (≤50 000 nuclei, 9 µL per well) and 10 µm well‐specific barcoded RT primers (1 µL per well) (Table S3, Supporting Information) were distributed to each well of the 96‐well plate and stirred gently with the pipette tip. The reaction mix was incubated with the thermal cycling: (8 °C for 12 s, 15 °C for 45 s, 20 °C for 45 s, 30 °C for 30 s, 42 °C for 2 min) x 10 cycles, 42 °C for 45 min. After the reaction, all nuclei were collected, mixed, and washed using PBST (PBS, 0.05% Tween 20) three times to remove the residual primers. After washing, nuclei were suspended in TdT mixture (100 000‐1 000 000 nuclei per reaction, 39 µL nuclei in PBST, 5 µL 10 x TdT buffer (NEB), 5 µL CoCl_2_ (NEB), 0.5 µL 100 mm dATP (Invitrogen), 0.5 µL TdT enzyme (NEB)). The TdT reaction mix was incubated at 37 °C for 30 min. After reaction, nuclei were washed using PBST three times. The nuclei were counted and diluted to 2000–8000 nuclei µL^−1^ using OptiPrep (Stem Cell). DNA extension reaction mixture was prepared (for 80 µL): 40 µL ddH2O, 16 µL thermopol buffer, 6 µL 10 mm dNTP, 6 µL BST 2.0 Warmstart (NEB), 6 µL RnaseH (NEB), 6 µL USER (NEB). Nuclei, 2 x DNA extension reaction mixture (M20 Genomics, VITAPilote‐EFT1200 kit), and barcoded beads were encapsulated into droplets using the microfluidic platform as previously described.^[^ ^11^ ^]^ All the required reagents for droplet reaction can be ordered from M20 Genomics company. The flow rates: 200 µL h^−1^ for nuclei/reaction mixture; 500 µL h^−1^ for oil; 50 µL h^−1^ for bead. The mean value of droplet volume is 0.48 nL (mean diameter is 96.9 µm). The droplets (20–50 µL per tube) were incubated at 37°C for 1 h, 50 °C for 30 min, 60 °C for 30 min, 75 °C for 20 min. Then the droplets were broken by mixing with equal amounts of 20% PFO (1H,1H,2H,2H‐Perfluoro‐1‐octanol, Sigma). The supernatant was collected after centrifugating and purified with 1.2 x DNA Clean Beads (Vazyme) and eluted in 40 µL ddH_2_O. Two rounds of PCR were performed to amplify cDNA and add sequence adapters (Table S3, Supporting Information). The amplified libraries were purified with 0.8 x DNA Clean Beads and quantified using Qubit (Invitrogen). Circularization was performed to obtain a sequencing nanoball library for MGI DNBSEQ using VAHTS Circularization Kit for MGI (Vazyme, NM201). Library sequencing was performed using DNBSEQ‐T7 with paired‐end reads of 100 or 150 bp.

### Species‐Mixing Experiment Data Processing

First, primer sequences and extra bases generated by the dA‐tailing step were trimmed in raw sequencing data. Then UFI (8 nts) and cell barcode (30 nts) were extracted from each read1, and 10 nts prebarcode was extracted from read2 and merged the sequenced barcodes that were uniquely assigned to the same accepted barcode with a Hamming distance of 2 nts or less. Next, read2 was used to generate the gene expression matrix with the STARsolo module in STAR (2.7.10a) with reasonable parameters. To determine the number of nuclei in each sample, the scattergram of log10(genes) was plotted for each possible barcode and used the position of the minimum with the highest value of log10(genes) as the threshold: only barcodes with the number of genes above this threshold were used for downstream analysis. Barcodes with more than 25% of detected UFIs belonging to other species were considered doublets/mixed. The remainder were assigned to either humans or mice. The FASTQ files were obtained for HEK293T and NIH/3T3 sequencing with 10x Genomics Chromium version 3.1 on their dataset page and VASA‐drop from GEO (GSM5369496). For gene detection saturation, the top 50 cells with the most read sequenced were used. Down sampling and gene counting were carried out on the bam file, in which only uniquely mapped genes were involved. For gene body coverage, RSeQC was used to calculate read count coverage, and the percentage of covered reads among total reads was used for the plotting.

### Tissue Sample Data Preprocessing

For each sequencing library, the poly‐A tail was trimmed from each raw sequencing read using Cutadapt. Subsequently, real cells were identified based on the number of reads per cell, utilizing a manually defined minimal read cutoff determined by the results of the UMI‐tools whitelist function. Reads were then aligned to the GRCh38 reference genome using the STAR 2‐pass mode, and only uniquely mapped reads were retained. Each read was assigned to its corresponding gene using the “gene” tag within the GRCh38 GTF by employing feature Count. The digital gene expression (DGE) was generated using the UMI‐tools count function. Batches were defined as each sequencing library for each patient. For each batch, quality control (QC) was performed using Scanpy. Low‐quality cells or potential doublets were removed by applying a manually defined cutoff for the number of UFIs and genes per cell, and genes expressed in less than 0.25% of the cells were also removed.^[^ ^72^ ^]^

### Cell Type Annotation

Expression analysis was performed using Scanpy. The cells from each patient were normalized and scaled. PCA was calculated and corrected using Harmony to remove potential batch effects, with the sequencing library set as the batch label. The top 40 principal components (PCs) were used to construct a nearest neighbor graph, with the n_neighbors parameter set to 10. Cell clustering was performed using the Leiden algorithm with a resolution of 0.4. The resulting clustering results were visualized using UMAP. Manual annotation of each cell cluster was carried out using canonical markers. The canonical markers were obtained from several high‐quality research papers, and the full list of these canonical markers is provided in Table S2 (Supporting Information). For the annotation of each cell cluster, the gene intersection of these canonical markers and the top 100 markers identified by the Scanpy rank_genes_groups function was used.

### Correlation Analysis Between Microwell‐Based scRNA‐Seq and snHH‐Seq Profiles

To correlate the snHH‐seq with the microwell‐based scRNA‐seq, their gene expression profiles were transformed into pseudo‐bulk expression profiles. For each broad cell type, their gene expression data was first extracted and calculated the CPM for each cell (nucleus) with the `*NormalizeData*` function (normalization method = “RC”; scale factor = 1e6) in the Seurat R package. Then, the CPMs for each gene were averaged row‐wise (across each cell (nucleus)) for each broad cell type separately. The resulting pseudo‐bulk expression profiles for both snHH‐seq and microwell‐based scRNA‐seq were then log2‐transformed. In this analysis, protein‐coding genes and long noncoding RNAs (lncRNAs) were focused. The complete human gene set was retrieved through the Ensembl database (GRCh38.p13) with the *BiomaRt* R package. In addition, all the protein‐coding genes and lncRNAs that we used had a mappability score >0.9. The mappability scores for GRCh38 were calculated by GenMap v1.3.0, following the instructions on its Github page (https://github.com/cpockrandt/genmap). The Spearman correlation between the Microwell‐based scRNA‐seq and the snHH‐seq profiles was then computed separately for protein‐coding genes and lncRNAs. The residual for each gene was calculated by using the “*resid*” function in R, after fitting a linear model to the snHH‐seq and microwell‐based scRNA‐seq data with the formula “*lm (microwell‐based scRNA‐seq – snHH‐seq ∼ 0)*”. The divergent genes were defined as the genes with residual values greater than 97.5th or less than the 2.5th percentile.

### Relationship Between Poly‐A Content and Gene Length

A poly‐A unit as one stretch of at least 20 consecutive adenine “A” bases in a gene's sequence was defined. The *BSgenome.Hsapiens.UCSC.hg38* R package was used to search such units for each gene in the human genome. The total poly‐A units with the gene length for microwell‐based scRNA‐seq and snHH‐seq separately using the *ggplot2* R package were then plotted.

### Mapping Microwell‐Seq and snHH‐Seq Cell Annotation with a Random Forest Classifier

To further validate the snHH‐seq method, Here, wanted to see whether the major cell‐intrinsic programs can be well preserved between the well‐validated microwell‐based method and the snHH‐seq method. For this aim, a multiclass random forest classifier on the snHH‐seq data and its cell annotation with the “*randomForest*” function from the *randomForest* R package were trained. Then the “predict” function was used to reannotate the microwell‐based scRNA‐seq data and compare the prediction with the original annotation. The resulting confusion matrix was plotted with the “*pheatmap*” function (scale = “row”) from the *pheatmap* R package. To train the random forest model, the top 3000 highly variable genes that were derived with the “*FindVariableFeatures*” function (“vst” as the selection method) from the *Seurat* R package were used. In addition, only shared broad cell types between the two methods were included.

### Comparison of Tissue Dissociation‐Induced Stress Signature Scores

According to a previous study, tissue dissociation is reported to induce gene expression changes which may affect the results of single‐cell RNA‐seq projects.^[^ ^73^ ^]^ With the published set of genes that are likely to be affected by dissociation, the dissociation signature for each shared broad cell type in the microwell‐based scRNA‐seq and snHH‐seq dataset with the “*AddModuleScore*” function in the *Seurat* R package was scored. The input gene expression profiles for both microwell‐based scRNA‐seq and snHH‐seq were normalized with the “*NormalizeData*” function in the *Seurat* R package with all default settings and then log‐transformed.

### Comparison of Transcript Fractions Mapped to Different Types of Genes

To compare the microwell‐based scRNA‐seq and snHH‐seq profiles, the distribution of transcript fractions that were mapped to different types of genes for the two methods was plotted. all the detected genes were divided into five broad groups: protein‐coding genes, transcription factors, lncRNAs, non‐polyadenylated genes, and short noncoding RNAs (sncRNAs). The protein‐coding gene, sncRNA, and lncRNA annotations were retrieved from the GRCh38 GTF file. The set of transcription factors was retrieved with the “dorothea_hs” function in the Dorothea R package. The set of non‐polyadenylated RNAs was downloaded from the supplementary data provided by Yang et al.^[^ ^9^ ^]^ The plots were generated with the *ggplot2* R package.

### CNV Inference and CNV Score

The InferCNV package was applied to infer the CNVs. For each patient, inferCNV was executed with a default cutoff of 0.1, utilizing annotated nonepithelial cells (non‐glial cells for Glioma) such as macrophages, endothelial cells, and stromal cells from the corresponding tissue. To calculate the CNV score for each cell, the inferCNV results were imputed by incorporating the CNV results of the cell itself and its eight adjacent cells, as determined by the neighborhood graph of clustering results from each patient. The imputed CNV results were then categorized into three levels (2/1/0/1/2) based on predefined intervals: [0.9, 0.95, 0.98, 1.02, 1.05, 1.1], [0.85, 0.925, 0.97, 1.03, 1.075, 1.15], or [0.8, 0.9, 0.96, 1.04, 1.1, 1.2], considering the distinct upper and lower limits of inferCNV results. Finally, the CNV score for each cell was determined by calculating the average of the imputed CNV scores across all genes associated with that particular cell.

### Malignant Cell Identification

All analyses were performed using the Python package Scikit‐learn. For each tissue, the imputed inferCNV results obtained from the annotated epithelial cells (or annotated glial cells for Glioma) of each patient were extracted and merged. Dimensionality reduction was then conducted to convert CNV scores to 50 PCs using the PCA function. The resulting cell‐by‐PC matrix was then employed for hierarchical clustering, using the pdist and linkage functions. Hierarchical clusters were visualized using the dendrogram function, and the final clusters were defined using the fcluster function. Clusters characterized by relatively low CNV scores and a mixture of cells from different patients were identified as nonmalignant cells for the respective tissue, while the remaining cells were classified as malignant.

### Integration of Malignant Cells and Nonmalignant Cells

The integration of malignant cells was performed using Harmony (*θ* = 6) with the cancer type specified as the batch label. Before integration, tissue‐specific genes were identified using Seurat's FindConservedMarkers function (logFC > 0.25, *p* < 0.05, min.pct > 0.25) and subsequently removed from the expression matrix. The clustering step was carried out using the following parameters: pc = 30, resolution = 0.4, and neighbor = 30. For the integration of nonmalignant cells, including endothelial cells, stromal cells, and monocytes, the Seurat SCTransform method was employed. Cancer type was designated as the batch label, and the analysis utilized pc = 30 and resolution = 0.5 as the settings.

### Exon Percentage

Exon‐only DGE was generated by using the “exon” tag in the feature count step. The exon percentage for each gene was calculated by dividing the UFI count within the original DGE by the UFI count within the exon‐only DGE.

### Cell Cycle Assigning

Proliferated non‐malignant epithelial cells were identified by integrating all nonmalignant epithelial cells using Harmony (*θ* = 3) with the following settings: pc = 30, resolution = 0.2, and neighbor = 30. As previously mentioned, tissue‐specific genes were removed before the integration process. To assign cell cycle phases for both malignant and non‐malignant epithelial proliferated cells, the Scanpy score_genes_cell_cycle function was utilized.

### Differentially Expressed Gene Identification

Differentially expressed genes (DEGs) were identified using the Seurat FindMarker and FindConservedMarker functions.

- Malignant cell DEGs for each cancer were identified using FindMarker (pct > 0.1, adj.*p* < 0.01, logFC > 1, pct.1 – pct.2 > 0) with the removal of patient and sample (tumor or NAT)‐specific genes (FindConservedMarker, pct > 0.25, *p* < 0.05, logFC > 0.5, pct.1 – pct.2 > 0.1).
- For each cancer subcluster, marker genes were identified using Seurat FindMarker with the following cutoff: pct > 0.25, adj.*p* < 0.01, logFC > 0.5, pct.1 – pct.2 > 0.
- Cell cycle phase‐specific DEGs for each tissue were identified using FindMarker (pct > 0.25, adj.*p* < 0.01, logFC > 1, pct.1 – pct.2 > 0.1) with the removal of patient and sample (tumor or NAT)‐specific genes (FindConservedMarker, pct > 0.25, *p* < 0.05, logFC > 0.5, pct.1 – pct.2 > 0.1). Furthermore, only DEGs that were also up‐regulated compared with quiescent cells were retained (FindConservedMarker, pct > 0.25, *p* < 0.05, logFC > 0.5, pct.1 – pct.2 > 0.1).
- DEGs between malignant ciliated‐like cells and non‐malignant ciliated cells in the lung were identified using FindConservedMarker (pct > 0.25, *p* < 0.05, logFC > 0.5, pct.1 – pct.2 > 0.1).

### Gene Regulatory Network Inference

The gene regulatory network was inferred using pySCENIC with default settings. The subsampled DGE contained 1000 cells per cancer type from 10 pan‐cancer groups, and 1000 cells per patient for nonmalignant epithelial cells were used as input. Differentially expressed transcription factors were identified using the Seurat FindMarker function.^[^ ^74^ ^]^

### Somatic Mutation Calling

To examine the difference in somatic mutation signatures among different lineages, 1000 stromal, epithelial, and cancer cells per patient to avoid the effect of different sample sizes were randomly sampled. Additionally, 1000 cells per cancer type from ten pan‐cancer groups detected by Leiden were sampled and used for comparison. The practical guidelines provided by Mutect2 (Somatic short variant discovery (SNVs + Indels) – GATK (broadinstitute.org)) to call somatic mutations were applied. Basic preprocessing included adding read groups, mark duplicates, and split reads that contained Ns in their CIGAR strings, and the default setting was applied. Several known mutation reference sites from the GATK resource bundle (https://gatk.broadinstitute.org/hc/en‐us/articles/360035890811‐Resource‐bundle) and dbSNP were provided for BQSR.^[^ ^75^ ^]^ The tumor‐only mode for somatic calling with hg38 was used as the genome reference and provided a 1000 panel of normal samples. A strict filtering strategy was applied to select good‐quality somatic mutations; specifically, orientation bias artifacts and contamination were excluded, the sum depth of the reference allele and alternate allele was larger than ten, and at least three alternate alleles were detected. Funcotator was applied to annotate the detected somatic mutations. The annotated maf file was analyzed by maftools in R. On average, 440 mutated genes per 1000 malignant cells per patient and 308.5 mutated genes per 1000 nonmalignant epithelial cells per patient with C to T as the most frequent SNV class and NEAT as the most mutated gene were counted (Figure S8d,e, Supporting Information).

### Splicing Transcript Analysis and Generation of the Pseudo‐Transcript Expression Matrix

The alignments from STAR were used to collect and quantify isoform proportions for cancer genes (CSC gene source: cosmic census^[^ ^76^ ^]^) using DICEseq, which is a Bayesian method based on a mixture model whose mixing proportions represent isoform ratios. For cancer cells, the Leiden groups detected in pan‐cancer analysis were used as pseudo‐bulk RNA‐seq groups. With 7 cancer types in total, for each cancer type, there were ten pseudo‐bulk RNA‐seq groups. Matched NAT samples were calculated per patient base. There were two types of isoform proportion matrix results, cancer type, and epithelium type, and the matrix was the isoform proportion for the pan‐cancer cluster or epithelial group. Pseudo‐transcript expression based on the isoform proportion and raw expression matrix by multiplying the related portion in that group was generated.

### Somatic Mutation Analysis

The resulting annotated file was stored in mutation annotation format (maf). The mutation count matrix was extracted by mutCountMatrix() of maftools. PCA components were calculated from the integrated mutation count matrix of patients and used to perform correlations. To select commonly mutated genes among different cancer types, A Wilcoxon test was performed on variant counts of the malignant group, stromal group, and nonmalignant epithelial group to obtain significantly mutated genes with logfold changes >20. Four inputs included the number of mutation loci per gene (count per gene) per sample and the depth of mutations per gene (depth per gene) per sample by patient level or per cluster level. Nonsynonymous mutations and synonymous mutations were both included. Synonymous mutations were included by using argument vc_nonSyn in read.maf to include different kinds of variant classifications (“3′UTR”, “RNA”, “5′Flank”, “Missense_Mutation”, “IGR”, “Silent”, “5′UTR”, “Nonsense_Mutation”, “Splice_Site”, “Translation_Start_Site”, “Nonstop_Mutation”, “Intron”). Sixteen common significantly mutated genes were detected with four approaches (Figure S8a, Supporting Information). Overall, intronic mutation and bases substitution C>G ranked the main variant classification and SNV class. Median variants per sample in cancer was 440, higher than 308.5 in epithelial cells (Figure S8b, Supporting Information).

### Malignant Cell Subclone Identification

For the case study of the COAD_p1 sample, the R package Numbat to identify malignant cell subclones was utilized. Initially, SNPs were piled up and phased using Cellsnp‐lite and Eagle2 with numbat default settings, except for minMAF = 0.1 and minCOUNT = 20 to increase the accuracy of SNP identification. The input consisted of a BAM file containing malignant and non‐malignant epithelial cells from COAD_p1 as well as the 1000 Genome hg38 SNP VCF file, 1000 Genome hg38 phasing panel file, and Eagle2 hg38 genetic map. CNV‐based subclones were inferred using the run_numbat function with endothelial cells, macrophages, fibroblasts, and immune cells from COAD_READ as a reference.

## Conflict of Interest

G.G., Y.W., and H.C. are inventors of a patent application covering the method. Multiple authors are involved in the commercialization of the technique and engage with M20 Genomics, Inc. (Y.W. and G.G. are cofounders, equity holders, and consultants; J.C., T.Z., Y.L., and H.C. are employees). The remaining authors declare no competing interests.

## Author Contributions

H.C., X.F., J.S., Q.Z., L.X., and J.C. contributed equally to this work. G.G., Y.W., and H.C. conceived the study and designed the project. H.C., M.J., and Y.L. performed snHH‐seq experiments. X.F. designed and built the upstream and downstream snHH‐seq analysis platform. J.S. performed upstream processing. X.F. and J.S. performed downstream pan‐cancer expression analysis, constructed an annotated database, and developed a versatile specific snHH‐seq data analysis pipeline. J.S., J.C., Y.M., L.M., P.Z., Y.F., T.Z., and J.W. performed benchmark analysis. Q.Z., L.X., Z.L., Z.C., Y.C., C.Y., D.C., L.J., F.Y., W.L., G.C., H.S., C.W., and T.L. provided clinic samples and diagnosis. Y.W. and Y.L. constructed the microfluidic platform. G.G., Y.W., and X.H. supervised this project. H.C., X.F., J.S., and J.W. wrote the manuscript; all authors have revised and approved the final manuscript.

## Supporting information

Supporting Information

Supplemental Table 1 Patient information

Supplemental Table 2 annotation

Supplemental Table 3 oligo

Supplemental Table 4 abbreviation

Supplemental Table 5 figure source data

## Data Availability

The data that support the findings of this study are openly available in the Genome Sequence Archive in the National Genomics Data Center, China National Center for Bioinformation/Beijing Institute of Genomics, Chinese Academy of Sciences at https://ngdc.cncb.ac.cn/gsa‐human, reference number HRA003939. The data deposited and made public are compliant with the regulations of the Ministry of Science and Technology of China. Processed count matrices and cell annotations are provided on the figshare website. Other raw data are available in Gene Expression Omnibus under accession number GSE237166. The analysis code customized for snHH‐seq sequencing data is available at https://github.com/ggjlab/HH‐seq/.
