# Supplementary material for: Pan‐Cancer Single‐Nucleus Total RNA Sequencing Using snHH‐Seq
Source: Adv Sci (Weinh). 2023 Nov 27;11(5):2304755. doi: 10.1002/advs.202304755 (PMC10837386; doi:10.1002/advs.202304755)
Supplement: Supplementary file 1 — Supporting Information [file ADVS-11-2304755-s006.pdf]

ADVANCED SCIENCE
Open Access

Supporting Information

for *Adv. Sci.*, DOI 10.1002/advs.202304755

Pan-Cancer Single-Nucleus Total RNA Sequencing Using snHH-Seq

Haide Chen, Xiunan Fang, Jikai Shao, Qi Zhang, Liwei Xu, Jiaye Chen, Yuqing Mei, Mengmeng Jiang, Yuting Wang, Zhouyang Li, Zihang Chen, Yang Chen, Chengxuan Yu, Lifeng Ma, Peijing Zhang, Tianyu Zhang, Yuan Liao, Yuexiao Lv, Xueyi Wang, Lei Yang, Yuting Fu, Daobao Chen, Liming Jiang, Feng Yan, Wei Lu, Gao Chen, Huahao Shen, Jingjing Wang, Changchun Wang*, Tingbo Liang*, Xiaoping Han*, Yongcheng Wang* and Guoji Guo**

Supporting Information

Pan-cancer single-nucleus total RNA sequencing using snHH-seq

Haide Chen, Xiunan Fang, Jikai Shao, Qi Zhang, Liwei Xu, Jiaye Chen, Yuqing Mei, Mengmeng Jiang, Yuting Wang, Zhouyang Li, Zihang Chen, Yang Chen, Chengxuan Yu, Lifeng Ma, Peijing Zhang, Tianyu Zhang, Yuan Liao, Yuexiao Lv, Xueyi Wang, Lei Yang, Yuting Fu, Daobao Chen, Liming Jiang, Feng Yan, Wei Lu, Gao Chen, Huahao Shen, Jingjing Wang, Changchun Wang*, Tingbo Liang*, Xiaoping Han*, Yongcheng Wang*, and Guoji Guo**

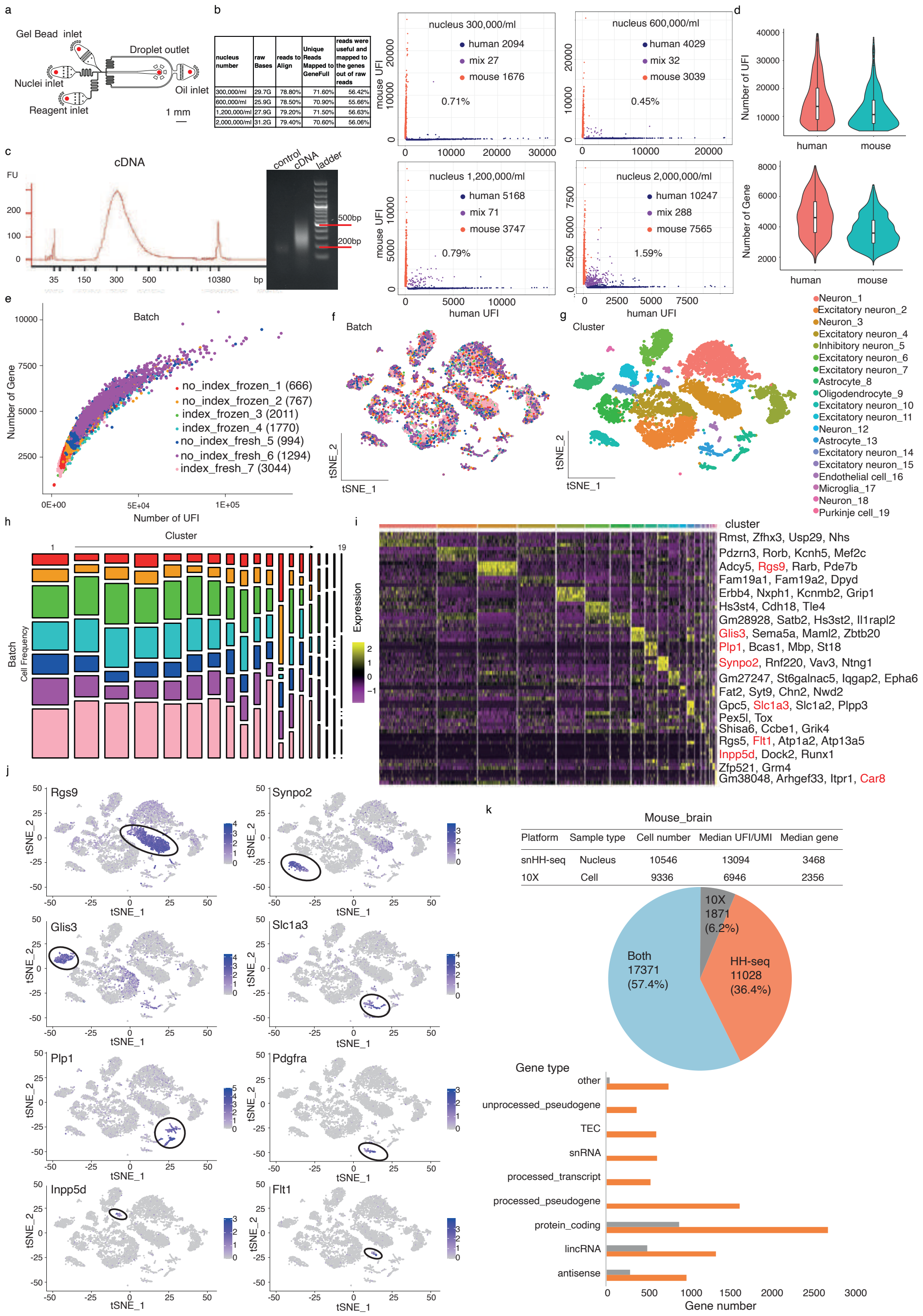

Supplementary Figure 1. Validation of the snHH-Seq Platform

a) Design of the device for nuclei, bead and mix reagents encapsulation. **b**) Human–mouse mix test with nuclei overloading. The fractions of reads used in gene mapping (left), the scatter plot of human–mouse mix test (right) with different nuclei overloading

(8 pre-index barcodes, load with ~300,000 nuclei/ml to ~2,000,000 nuclei/ml, 150 µL droplet). **c**) Size distribution of enriched cDNA (human-mouse mix) obtained using snHH-seq. 2100 bioanalyzer (**left**), gel electrophoresis (**right**). **d**) Distribution of UFI (left) and Gene (right) detected in 293T (human) and 3T3 (mouse) nuclei (for Figure 1d). **e**) UFI/gene plot for mouse brain with different batches. No_index, RT primer without barcode; index, RT primer with barcode; frozen, nuclei isolated from frozen sample; fresh, nuclei isolated from fresh sample. **f**)-**g**) t-SNE of mouse brain single nucleus profiles from different batches, colored by batch (**f**) and assigned cell subset signature (**g**). **h**) Cell type composition of mouse brain. Proportion of cells in each cluster in each of the 7 batches. **i**) Heatmap of top differentially expressed genes for mouse brain. Yellow corresponds to high-expression levels; purple and black correspond to low-expression levels. **j**) t-SNE maps of mouse brain with cells colored based on the expression of cell type markers. Gene expression levels are indicated by shades of purple. **k**) Percentage of genes detected in snHH-seq compared to 10X for mouse brain. 57.4% of the detected genes were shared between the methods; 36.4% were detected only in snHH-seq; and 6.2% were detected only in 10X Chromium (top). Counts of different RNA biotypes (bottom). 10X whole mouse brain dataset GSM6617915.

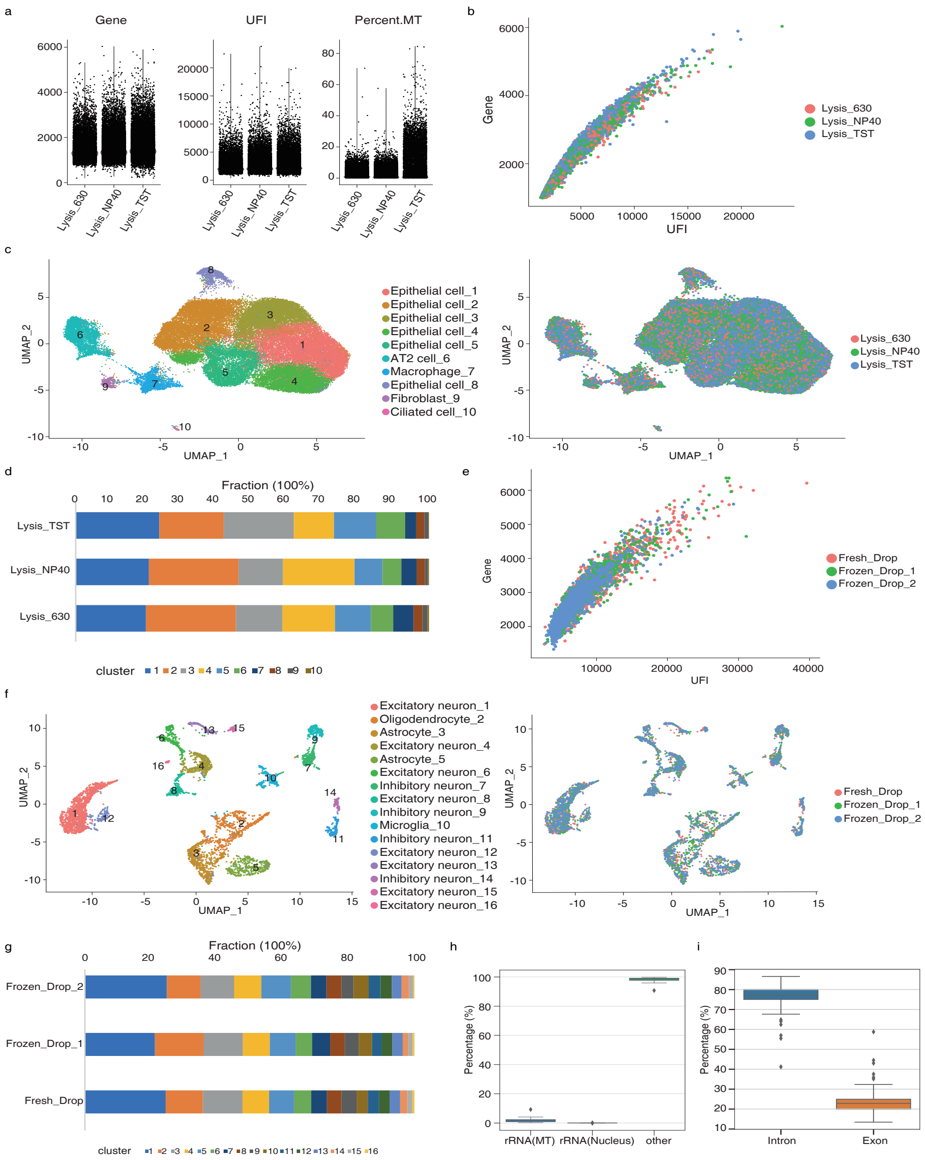

Supplementary Figure 2. snHH-seq performs in cancer samples

a) Distributions of gene, UFI and mt-gene detected in lung cancer (P7) with different lysis buffers. Lysis-630, 0.1% IGEPAL CA-630 with salts and Tris; Lysis-NP40, 0.1% NP40 with salts and Tris; Lysis-TST, Tween with salts and Tris. **b**) UFI/gene plot for lung cancer (P7) with different lysis buffers. **c**) UMAP of lung cancer (P7) single nucleus profiles from three buffers, colored by assigned cell subset signature (left) and buffer (right). **d**) Cell type composition of lung cancer (P7). Proportion of cells in each cluster in each of the three buffers. **e**) UFI/gene plot for brain cancer (part of Glioma

P2/P3/P4) with different protocols. Fresh-Drop, nuclei are not cryopreserved before drop procedure; Frozen-Drop, nuclei are cryopreserved before drop procedure. **f**) UMAP of brain cancer single nucleus profiles from different protocols, colored by assigned cell subset signature (left) and protocol (right). **g**) Cell type composition of brain cancer. Proportion of cells in each cluster in each of the two protocols. **h**) The percentage of rRNA UFI detected in snHH-seq. Box plots show median, quartiles, and whiskers at 1.5 times the interquartile range (IQR). **i**) The percentage of exon and intron detected in snHH-seq. Box plots show median, quartiles, and whiskers at 1.5 times the interquartile range (IQR).

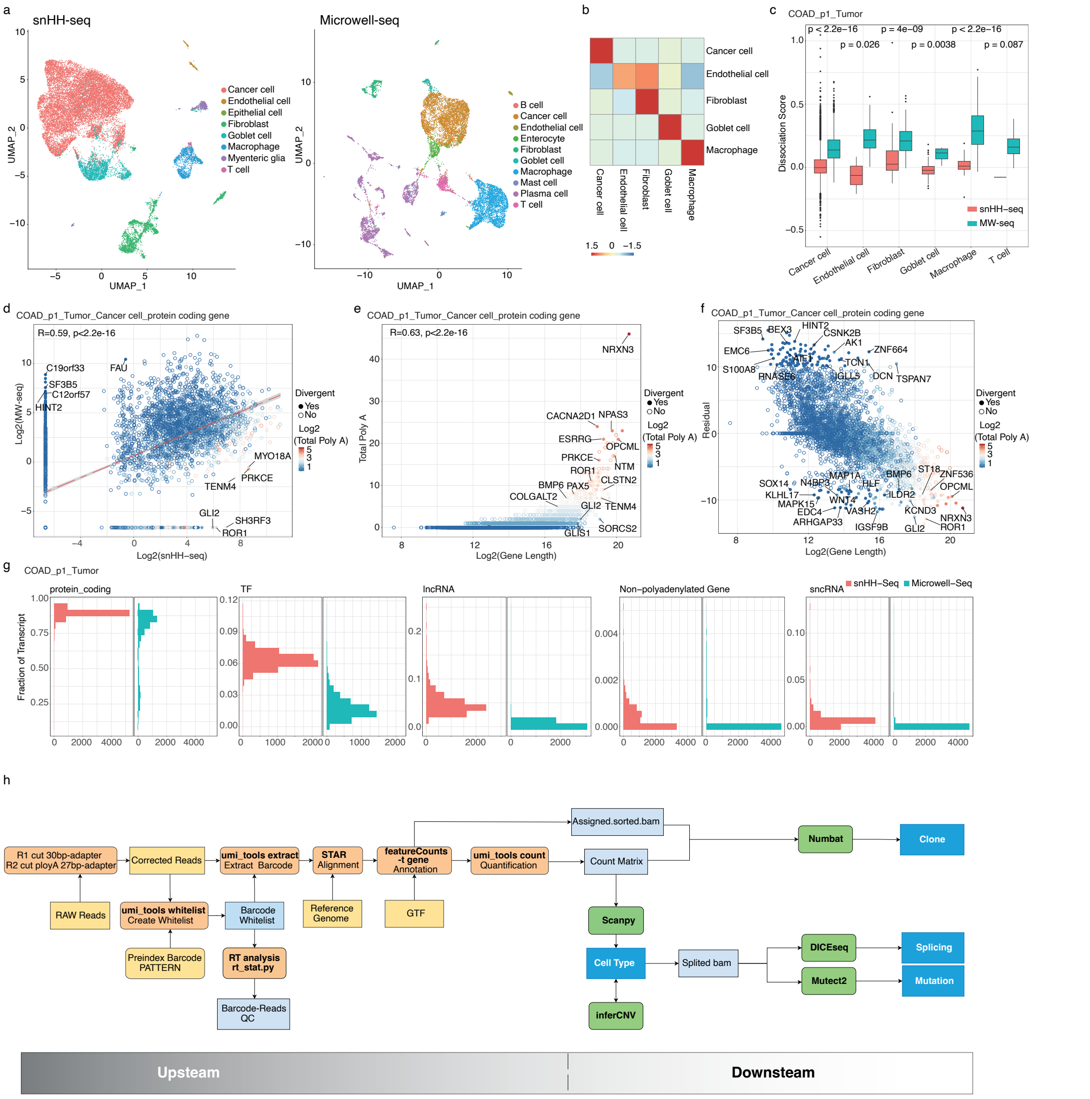

Supplementary Figure 3. Comparison of gene detection between snHH-seq and Microwell-seq

a) UMAP visualization of single nucleus profiles (snHH-seq, left) and single cell profiles (Microwell-seq, right) for COAD_P1. **b**) Cell-type correlation analysis matched cell types between snHH-seq and Microwell-seq for COAD_P1. **c**) Tissue dissociation expression signatures in scRNA-seq of COAD_P1_tumor. Scores [y axis, average background corrected log(TP10K+1)] of a dissociation-related stress signature in scRNA-seq (blue) and snRNA-seq (pink) profiles in each major colon cell type (x axis) are shown. Wilcoxon rank sum test, box plots show median, quartiles, and whiskers at 1.5 times the interquartile range (IQR). **d**) Divergent genes between cell

and nucleus profiles. Averaged pseudobulk expression of protein coding genes (dots) in COAD_P1_tumor cancer nuclei (x axis) and cells (y axis) is shown. Divergent genes are represented by black dot. The color scale shows the total length of polyA stretches. **e)-f)** Relation between gene expression differences in nuclei vs. cells, gene length and polyA stretches. (**e**) The number of polyA stretches (y-axis) and length (x-axis) of each gene. (**f**) Divergence (y-axis, residual of straight-line regression fit) between pseudobulk gene expression of single cell and single nucleus RNA-seq and gene length (x-axis) for each protein coding gene expressed in COAD_P1_tumor cancer cell in both datasets. **g)** Fraction of transcripts per biotype in snHH-seq compared to Microwell-seq for COAD_P1_tumor. **h)** Workflow for analyzing tumor samples.

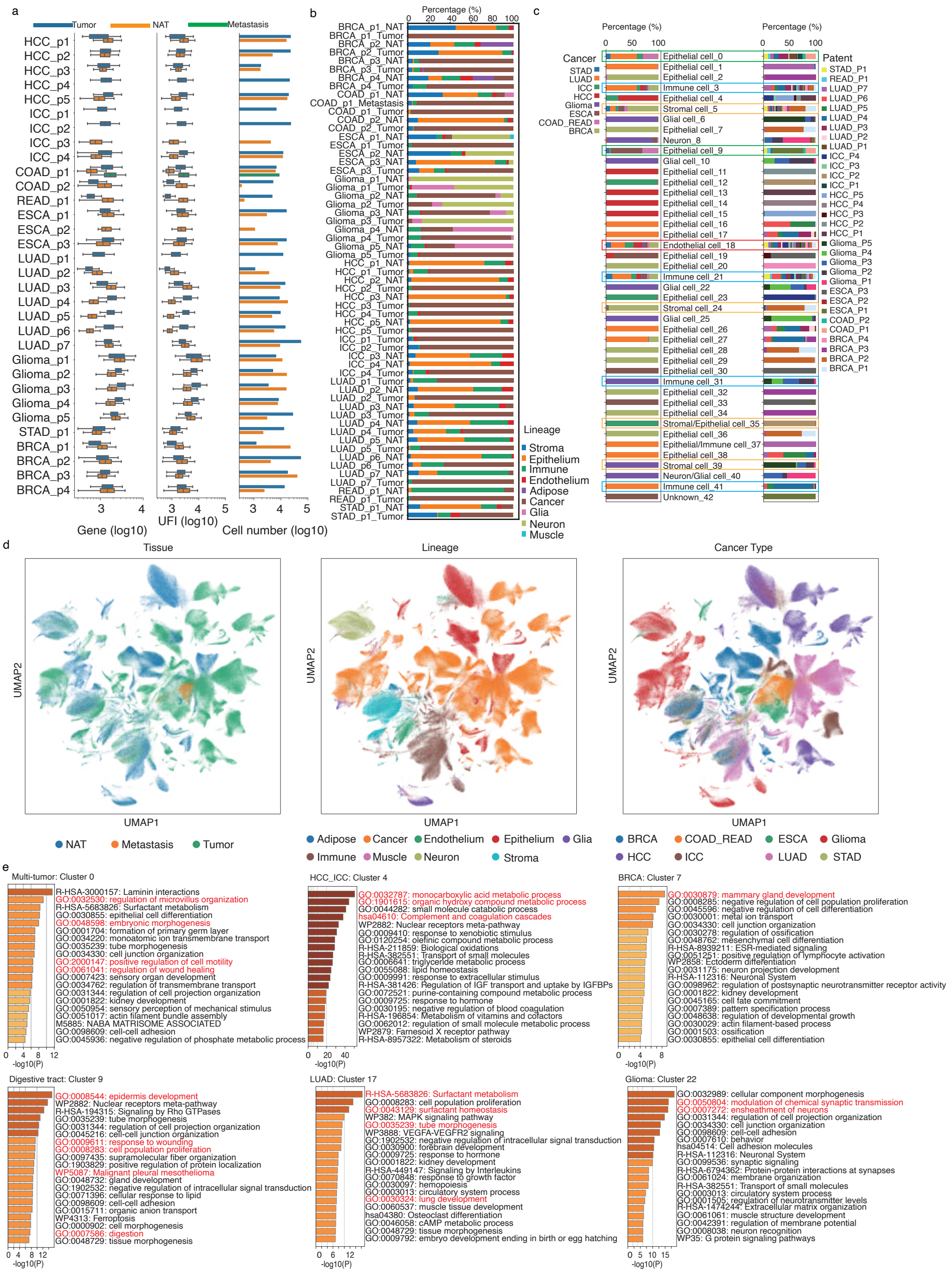

Supplementary Figure 4. Construction of the pan-cancer landscape using snHH-seq

a) Bar plots representing per patient from left to right: the number of genes, the number of UFIs, the number of cells. Box plots show median, quartiles, and whiskers at 1.5 times the interquartile range (IQR). **b)** The fraction of lineage per sample. **c)**The fraction of cells per tumor and per patient in each cell cluster (cell cluster in Figure 2c).

d) Uniform manifold approximation and projection (UMAP) embedding of cells from the 32 tumors analyzed in this study. Color-coded for tissue (left), lineage (middle) and cancer type (right). **e)** Gene ontology (GO) enrichment of cluster 0, 4, 7, 9, 17 and 22 (cell cluster in Figure 2c) using Metascape. (p values was calculated by the hypergeometric distribution, statistical test is one-sided, adjustments p values were made after p value is corrected by Benjamin & Hochberg multiple test).

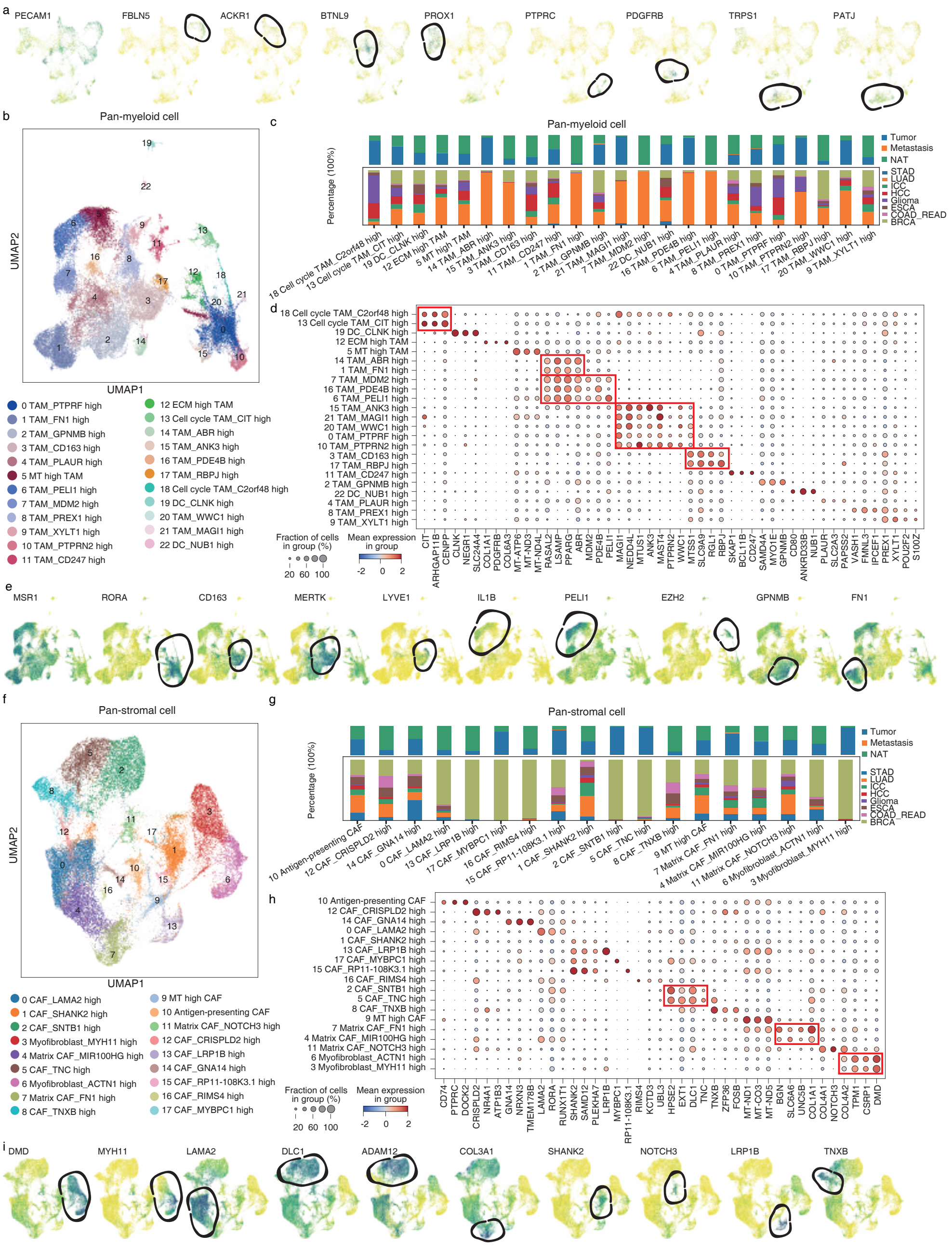

Supplementary Figure 5. Characterization of pan-cancer endothelial cell, myeloid cells and stromal cells

a) Marker genes expression for each cluster of tumor endothelial cells. **b)** UMAP of pan-cancer myeloid cells. **c)** Tumor distribution in each myeloid cell cluster. Color-coded for tumor. **d)** Marker genes expression per myeloid cell cluster. **e)** Marker genes expression for each cluster of myeloid cells. **f)** UMAP of pan-cancer stromal cells. **g)**

Tumor distribution in each stromal cell cluster. Color-coded for tumor. **h)** Marker genes expression per stromal cell cluster. **i)** Marker genes expression for each cluster of stromal cells.

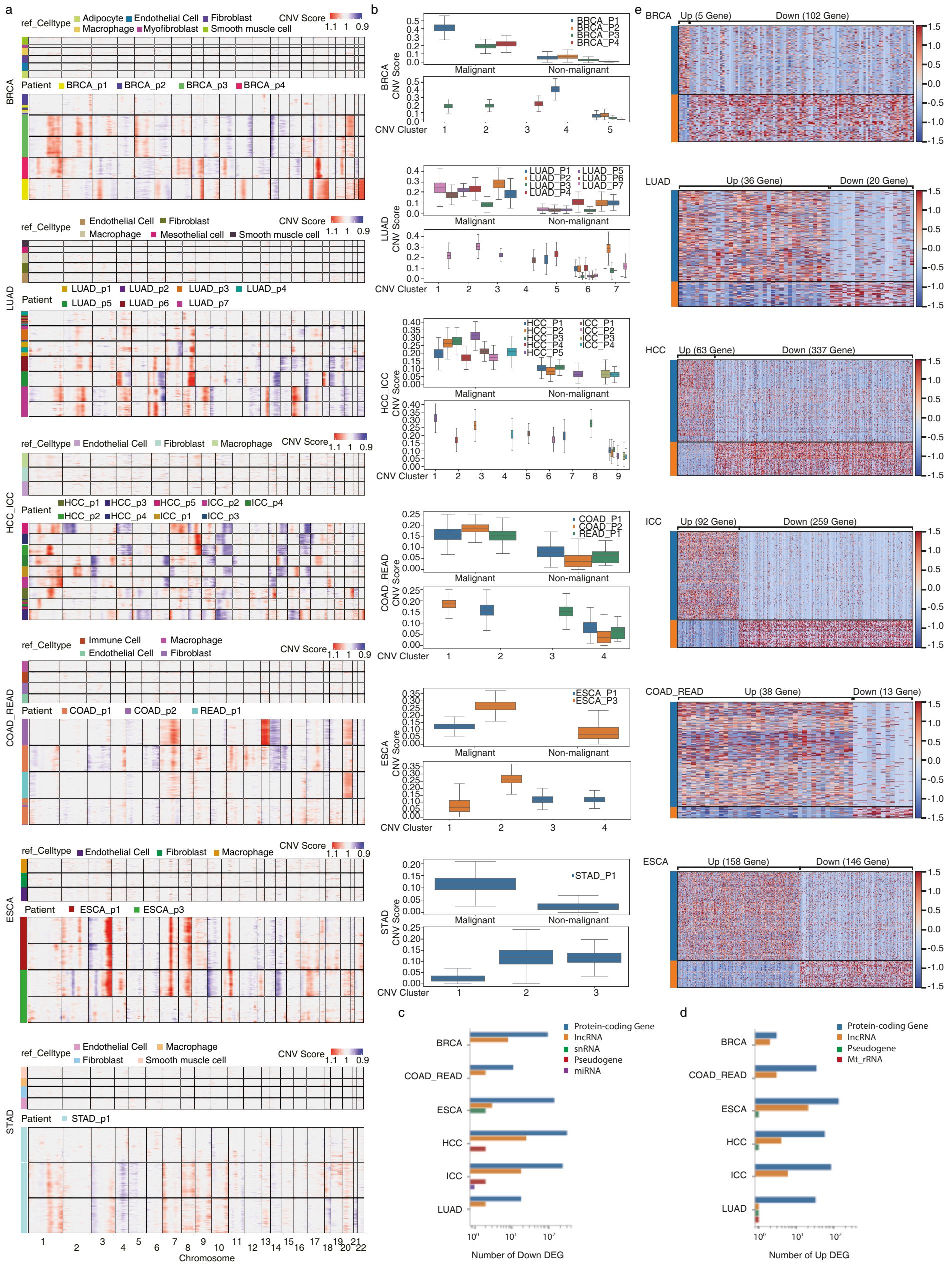

Supplementary Figure 6. CNV profiles inferred from snRNA-seq data for epithelial tumor

a) Hierarchical clustering results of CNV inferred from snRNA-seq data for epithelial tumor samples. Chromosomal amplification (red) and deletion (blue) are inferred in each chromosomal position (columns) across the single cells (rows). Top: reference cells not expected to contain CNVs, color-coded for cell type. Bottom: malignant and non-malignant epithelial cell, color-coded for patient. **b)** CNV score calculated by

inferCNV for epithelial tumors. **c)-d)** The number of down- (c) and up- (d) regulated genes between malignant and non-malignant epithelial cell for each cancer type. Color-coded for gene type. **e)** Heatmap of common DEGs for each cancer type.

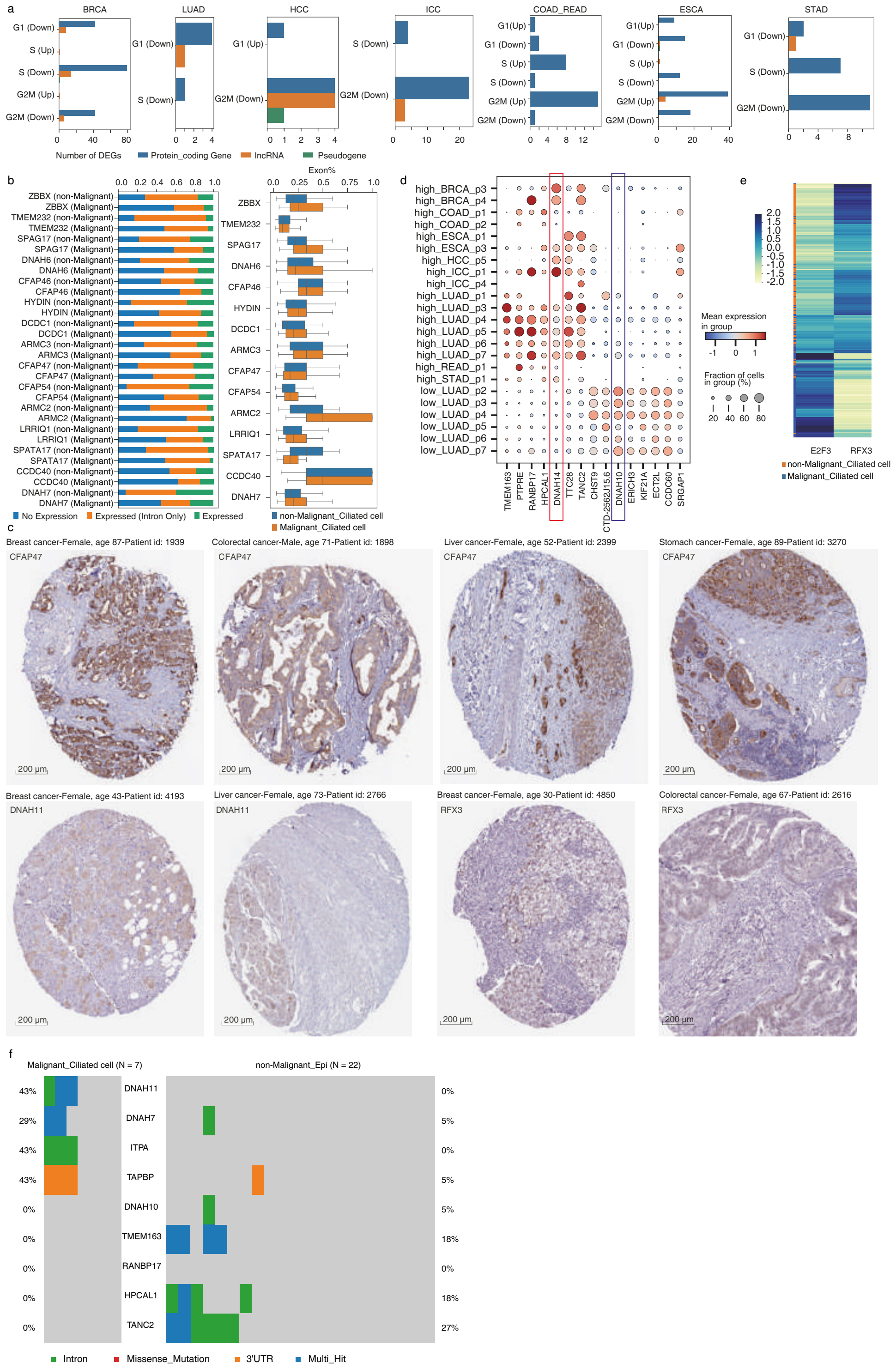

119

120 **Supplementary Figure 7. Patterns of proliferating malignant cells and ciliated-**

121 **like malignant cells**

122 **a)** The number of DEGs between proliferating malignant and non-malignant epithelial

cell in each cancer type. Color-coded for gene type. **b)** Expression proportions and exon proportions of selected cilia-related genes in non-malignant lung ciliated cells and ciliated-like malignant cells. **c)** Immunohistochemistry assay of cilia marker genes (CFAP47, DNAH11 and RFX3) in tumors (from Human Protein Atlas proteinatlas.org). **d)** DEGs between malignant ciliated-like cell and non-malignant lung ciliated cell. **e)** Differentially expressed transcriptional factors E2F3 and RFX3 between malignant ciliated-like cell and non-malignant lung ciliated cell. **f)** Mutation profiles of several ciliated-related genes and several DEGs between malignant ciliated-like cell and non-malignant lung ciliated cell, in malignant ciliated cell and non-malignant epithelial cell.

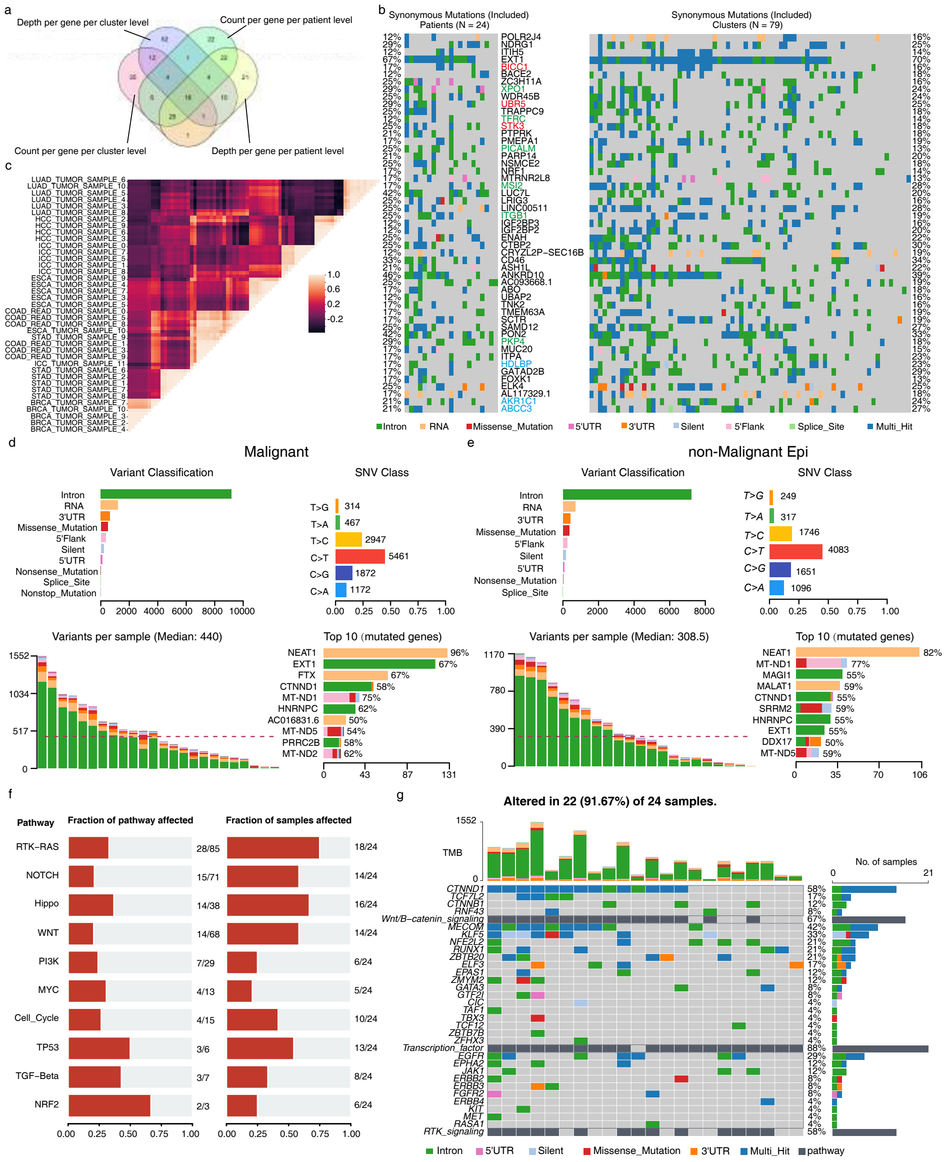

Supplementary Figure 8. Mutation characteristics of malignant epithelial cell

a) Intersection of 4 mutations profile matrix, count per gene indicates the number of mutation loci per gene, depth per gene indicates the depth of mutations per gene, per cluster level indicates 1000 cells sampled from annotated pan-cancer cluster, per patient level indicates 1000 cells sampled from individual patient. **b)** Co-Oncoplot of mutation counts include synonymous mutations between patient level and pan-cancer cluster level, 24 patient samples and 79 cluster samples in total (rows: commonly mutated gene, columns: samples of 1000-cell size), colored by mutation types. **c)** Correlation heatmap using principle components of mutation count matrix of all cancer types, mutations

profiles from same cancer type show strong correlation. **d)-e)** Summarization of detected mutation variants of malignant samples and non-malignant samples. Variant classification, SNV class, Variant counts per sample and top 10 mutated genes are shown. **f)** Well-known pathway affected in malignant cells. **g)** Most affected pathways including WNT, transcription factor and RTK-RAS and their affected mutated genes shown in 24 patient samples.

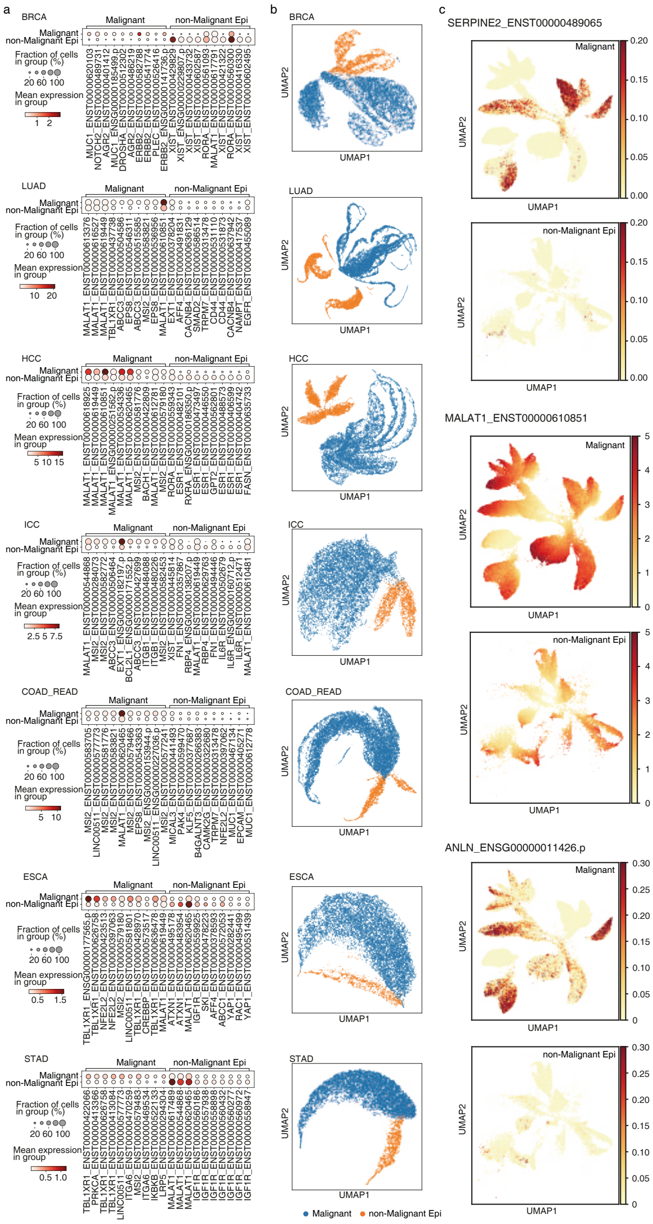

Supplementary Figure 9. Expression of cancer markers

a) Differential transcript expression for 7 cancer types (BRCA, LUAD, HCC, ICC, COAD_READ, ESCA and STAD) of known cancer markers. **b)** UMAP of pseudo transcript expression shows high distinction between malignant cells and non-malignant epithelium cells. **c)** Significant high expression of SERPINE2_ENST00000489065, MALAT1_ENST00000610851 and ANLN_ENSG00000011426.p shown in malignant cells and low expression in non-malignant epithelium cells.
